# The effect of development on cortical auditory evoked potentials in normal hearing listeners and cochlear implant users

**DOI:** 10.3389/fnhum.2025.1473365

**Published:** 2025-10-15

**Authors:** Eun Kyung Jeon, Carolyn Brown, Paul Abbas, Bruce Gantz

**Affiliations:** ^1^Department of Communication Sciences and Disorders, University of Iowa, Iowa, IA, United States; ^2^Department of Otolaryngology–Head and Neck Surgery, University of Iowa, Iowa, IA, United States

**Keywords:** cochlear implant, cortical auditory evoked potentials, P1-N1-P2 complex, acoustic change complex, developmental effects, pediatric cochlear implant users, normal hearing listeners

## Abstract

**Introduction:**

Cortical auditory evoked potentials (CAEPs), such as the P1-N1-P2 complex (onset response) and the acoustic change complex (ACC), provide insight into sound detection and discrimination. While their developmental trajectories are well documented in normal-hearing (NH) listeners, less is known about how these responses develop in pediatric cochlear implant (CI) users and how they are affected by background noise.

**Methods:**

CAEPs were recorded in quiet and +10 dB SNR noise conditions using long-duration vowel stimuli from 91 children and 11 adults with NH and 59 CI users (48 pre-lingually deafened children/young adults and 11 post-lingually deafened adults). Peak latencies (P1, N1, P2) and N1-P2 amplitudes were measured. Developmental effects were analyzed using linear regression, t-tests, and correlation analyses comparing child and adult waveforms.

**Results:**

Both onset and ACC responses were present across groups, with P1 latency decreasing significantly with age in NH and CI listeners. The ACC followed a similar developmental trajectory as the onset response but matured later, emerging reliably in adolescence. Noise delayed maturation, lengthened latencies, and reduced amplitudes, particularly for the ACC. CI users implanted before 3.5 years showed developmental patterns comparable to NH peers, though both onset and ACC responses were more affected by noise in CI users.

**Discussion/Conclusion:**

These findings demonstrate that early implantation supports the typical development of cortical auditory responses, underscoring the importance of neuroplasticity in pediatric CI users. However, the pronounced vulnerability of the ACC to noise highlights ongoing challenges in sound discrimination for CI users. CAEPs, especially ACC measures, may serve as objective markers of auditory maturation and could complement behavioral assessments in clinical practice.

## 1 Introduction

Cochlear implants (CIs) are electronic devices that bypass damaged parts of the cochlea to directly stimulate the auditory nerve, thereby restoring hearing. Since the first multichannel CI was implanted in a prelingually deafened child in 1987, CIs have become a highly successful tool for enabling speech perception in deaf children, greatly extending the candidacy for cochlear implants in the pediatric population (Carlson et al., 2015; Ching et al., 2017; Dettman et al., 2016, 2021; Hang et al., 2015; Hoff et al., 2019; Leigh et al., 2016; Park et al., 2019; Tomblin et al., 1999).

In the 1980s, most children who received a CI were 4–5 years old or older (Tomblin et al., 1999, 2005). Today, with newborn hearing screening, congenital deafness can be identified shortly after birth, allowing for early intervention with CIs within the first year of life. This early intervention and the improved outcomes with CIs have contributed to their widespread adoption globally. As of December 2019, approximately 736,900 registered devices have been implanted worldwide (National Institute on Deafness and Other Communication Disorders, 2024). This represents a significant increase from December 2012, when over 324,000 people worldwide had received CIs (Şahin et al., 2017), including more than 80,000 children (Kral and O’Donoghue, 2010). In the United States alone, roughly 118,100 devices have been implanted in adults and 65,000 in children.

Despite the consensus on the benefits of early cochlear implantation for better outcomes, auditory development in CI users inevitably lags that of children with normal hearing. Hearing development begins before birth, with fetuses responding to sounds as early as 24 weeks (Birnholz and Benacerraf, 1983). After birth, the central auditory pathways continue to mature over the first two decades of life, driven by auditory experiences (Moore D. R., 2002; Moore J. K., 2002). Many of the changes in the postnatal brain are experience-dependent; synapses that are frequently activated by auditory input will be strengthened, while those that are rarely activated will be lost. The central auditory system requires auditory experiences for typical development, including the proper maturation of neuronal activity, sound processing capabilities, and functional organization of the auditory cortex (Kral et al., 2000, 2001; Moore D. R., 2002; Moore J. K., 2002; Moore and Linthicum, 2007; Kral and Sharma, 2012).

Neuroplasticity, the brain’s ability to reorganize in response to environmental input or damage, is most pronounced during the first few years of life when synaptogenesis and synaptic pruning processes occur dynamically (Kral et al., 2001; Zhang et al., 2002; Nakahara et al., 2004; Sale et al., 2009; Pallas, 2001; Pascual-Leone et al., 2005). Damage to the cochlea can lead to sensorineural hearing loss (SNHL), resulting in the death of spiral ganglion cells (Hardie and Shepherd, 1999) and cochlear nucleus cells (Born and Rubel, 1988), leading to further developmental arrest of the central auditory system (Tremblay and Kraus, 2002; Turner, 2006).

However, leveraging the brain’s neuroplasticity through early electrical stimulation via CIs can mitigate these adverse effects. Animal studies show that electrical stimulation promotes an increased number of surviving spiral ganglion cells and neural responses in the inferior colliculus (Hyson and Rubel, 1989; Shepherd et al., 1999). Early implantation of CIs takes advantage of this neuroplasticity, allowing congenitally deaf children to develop auditory pathways and acquire speech and language skills. Behavioral studies have shown that the acquisition of spoken language is easier for children who are implanted earlier in life (e.g., Fryauf-Bertschy et al., 1997; Hassanzadeh et al., 2002; Harrison et al., 2005; Tomblin et al., 2005; Holt and Svirsky, 2008; Cole and Flexer, 2020; Ching et al., 2017; Dettman et al., 2016; Leigh et al., 2016; Kral et al., 2019).

Non-behavioral tools like auditory evoked potentials (AEPs) are valuable for studying central auditory development in children. The P1-N1-P2 complex is an obligatory cortical auditory evoked potential (CAEP) that assesses auditory processing at the cortical level in both normal-hearing (NH) individuals and cochlear implant (CI) users (Ponton et al., 1996b; Sharma et al., 1997). The P1-N1-P2 complex, typically elicited using brief stimuli such as clicks, pure tones, tone bursts, or short consonant-vowel syllables, indicates sound detection by the listener. The complex has a relatively long latency, reflecting the neural processing time from sound onset to the auditory brain, with neural generators located in thalamocortical pathways and primary and secondary auditory cortices. Each component has a different maturation period (Paus et al., 1999; Moore J. K., 2002; Stapells, 2002).

In NH listeners, the developmental patterns of the P1-N1-P2 response show P1 as the dominant component in young children, with N1 and P2 emerging with age (Ponton et al., 2002). The developmental effects on the morphology, latency, and amplitude of the P1-N1-P2 complex in NH listeners are well documented. In adults, all three components (P1, N1, P2) are present, with latencies between 50 and 250 ms after stimulus onset. P1, N1, and P2 typically occur at approximately 60 ms, 100 ms, and 175–200 ms, respectively, with the N1-P2 amplitude being the most dominant (Ponton et al., 1996a, 1996b, 2002; Sharma et al., 1997; Gilley et al., 2005; Kelly et al., 2005).

In infants and young children, P1 is the dominant component and is often the only identifiable peak, recorded between 150 and 350 ms after stimulus onset (Kurtzberg et al., 1984; Pasman et al., 1991; Ponton et al., 1996a, 1996b, 2002; Kelly et al., 2005). P1 amplitude in children is greater than in adults (Kushnerenko et al., 2002) and is present in about 95% of preterm babies at 35–37 weeks post-conceptual age (Pasman et al., 1991) and 97% of infants before 2 months (Kurtzberg et al., 1984). In young children, P1 is often followed by a broad negativity between 250 and 450 ms post-stimulus onset, considered an immature late cortical response (Kurtzberg et al., 1984; Pasman et al., 1991, 1999; Pang and Taylor, 2000; Kushnerenko et al., 2002; Sharma et al., 2002; Kelly et al., 2005; Sussman et al., 2008). Recent studies suggest that N1 and P2 components can be seen in infants with slower stimulation rates but with much longer latencies than in adults, overlapping with what is termed “immature negativity” (Small and Werker, 2012). N1 and P2 peaks typically begin to emerge around 7–8 years of age and are consistently recorded by 12–13 years. N1-P2 amplitudes increase with age (Ponton et al., 2000; Gilley et al., 2005; Wunderlich and Cone-Wesson, 2006), with Ponton et al. (2000) reporting that N1 amplitude becomes adult-like by 15–16 years of age.

Postnatal processes like myelination and synaptic pruning reduce neural travel time, reflected in decreased peak latencies with development (Ponton et al., 2000; Moore and Guan, 2001; Moore and Linthicum, 2007). P1 latency, less variable than N1 or P2 latencies, serves as a key measure for explaining the impact of development on the P1-N1-P2 complex. Sharma et al. (1997, 2002) identify P1 latency as a biological maturation marker of central auditory pathways.

Researchers have also successfully measured the P1-N1-P2 responses in pediatric CI users. Sharma et al. (2007) suggest that the onset P1-N1-P2 response can indicate whether the auditory nervous system is developing age-appropriately following (re)habilitation with a CI. They measured the P1-N1-P2 complex in 231 CI children (aged 1 to 20 years) at various times after implantation, dividing them into six groups based on age at implantation. Results indicate that P1 latencies in CI children decreased with increasing age, similar to NH children. Children implanted before 3.5 years showed P1 latencies within normal limits after 3 to 6 months of CI use, while those implanted at or after 7 years did not, regardless of CI use duration. Children implanted between 3.5 and 7 years showed variable results.

These findings suggest that early implantation (before 3.5 years) supports the normal development of central auditory pathways, while later implantation (after 7 years) is less effective. This aligns with research on speech and language development, highlighting that neuroplasticity is greatest up to 3 to 4 years of age (Huttenlocher and Dabholkar, 1997; Sharma et al., 2002, 2007; Harrison et al., 2005; Holt and Svirsky, 2008; Sharma and Campbell, 2011; Kral et al., 2019).

The Acoustic Change Complex (ACC), elicited by acoustic changes within a stimulus, indicates a listener’s sound discrimination ability. It shares morphological characteristics with the P1-N1-P2 complex but provides additional information on sound discrimination (Ostroff et al., 1998; Martin et al., 2008; Martin and Boothroyd, 1999). Martin and Boothroyd (2000) used an 800-ms synthetic vowel stimulus with a change at 400 ms, eliciting both the onset P1-N1-P2 response and the ACC, reporting that the ACC was successfully elicited by changes in sound level and formant frequency, with the greatest responses from stimuli with both changes. Recent studies further demonstrated the utility of the ACC for assessing cortical responses to changes in sound location and frequency. Fan et al. (2022) found that ACC could objectively evaluate spatial hearing by reliably detecting horizontal sound location changes, while Zhang et al. (2020) highlighted distinct cortical mechanisms for processing “what” (frequency) and “where” (location) sound information, with greater acoustic changes eliciting stronger and faster ACC responses.

Researchers have recorded ACC responses from CI users as well. Friesen and Tremblay (2006) found reliable ACC recordings from eight adult Nucleus CI users using speech syllables /si/ and /∫i/, with earlier latencies for /∫i/. Recorded ACCs using direct input to intracochlear electrodes, finding greater responses with larger spatial separation between electrodes. Kim et al. (2009) recorded EACCs from 10 CI users by changing current levels mid-stimulus, finding reliable responses to amplitude changes. Recent studies have shown that ACCs (or EACCs) can be reliably recorded in NH children and CI users (Martin et al., 2008; He et al., 2013; Martinez et al., 2013). He et al. (2013) recorded EACCs from 15 CI children with Auditory Neuropathy Spectrum Disorder (ANSD) using an 800-ms stimulus with various gap durations, finding larger EACC amplitudes with larger gaps. Martinez et al. (2013) recorded ACCs from NH and hearing-aid (HA) children aged 2–6 years using spectral changes, finding ACCs in most children, though morphology was poor in some HA users. More recently, Hu et al. (2024a) conducted simultaneous recordings of the P1-N1-P2 complex, ACC, and auditory steady-state responses (ASSR) in NH adults and CI users, focusing on onset/offset detection and left/right switching discrimination. These studies highlighted cortical responses to interaural-time-difference cues and CI stimulation artifacts, with Hu et al. (2024a) examining EEG artifacts and Hu et al. (2024b) exploring rate-dependent neural responses, advancing our understanding of auditory processing in CI users.

Although the ACC has been extensively used to assess auditory discrimination abilities across various listening groups and ages, studies on its developmental patterns are lacking, especially in children who grew up with NH or CIs. Additionally, studies comparing the maturation effects on the P1-N1-P2 and ACC responses in different listening environments, such as quiet and noise, are rare. Describing developmental changes in the ACC across different age groups in children with NH and CI users would enhance our understanding of cortical processing shown in auditory evoked potentials with maturation in both listening groups. Understanding these responses will provide a basis for clinical applications in estimating speech discrimination capacities in pediatric populations.

This study aims to investigate the development of the ACC and P1-N1-P2 complex in NH and CI listeners using long-duration speech stimuli in quiet and noisy conditions. We hypothesize that the ACC will follow similar developmental patterns to the onset P1-N1-P2 complex, with the maturation of the ACC being delayed relative to the onset P1-N1-P2. We also hypothesize that both onset P1-N1-P2 and ACC responses will be degraded (i.e., smaller amplitudes and longer latencies) in the presence of background noise across all age groups of NH listeners and CI users. This may lead to a delayed development of both onset P1-N1-P2 and ACC responses in noise compared to quiet listening conditions. Given that ACC responses are less robust and have smaller amplitudes than the onset P1-N1-P2, we expect ACC responses to be more affected by background noise. Additionally, we hypothesize that the developmental patterns of the onset P1-N1-P2 and the ACC in noise conditions will be similar in both NH listeners and CI users, but ACC responses will be more degraded in CI users compared to NH listeners in the presence of noise.

## 2 Materials and methods

### 2.1 Participants

Subjects were recruited for two listening groups: NH (Normal Hearing) and CI (Cochlear Implant). For NH listeners, ninety-one children and adolescents aged 3 to 19, and eleven adults aged 20 to 40 participated (mean = 28 years, SD = 6). All NH listeners had their hearing screened from 250 to 8,000 Hz in both ears. Table 1 shows the age distribution of participants in the NH group. Five children did not pass the hearing screening, had a cold, or did not complete the test; their data were excluded from further analysis.

**Table 1:** Age distribution of participants with normal hearing.

Age (in years)	3	4	5	6	7	8	9	10	11	12	13	14	15	16	17	18	19	20–40
*n* = 102	5	6	5	6	5	6	7	4	4	7	6	7	5	4	7	3	4	11

For CI users, a total of fifty-nine subjects participated. Forty-three pre-lingually deafened CI children and adolescents between the ages of 3 and 18, and five pre-lingually deafened CI adults between the ages of 20 and 30 participated (mean = 25, SD = 3). Additionally, eleven post-lingually deafened CI adults between the ages of 27 and 63 (mean = 50, SD = 14) participated in this study. All CI users were recruited from the University of Iowa Hospitals and Clinics (UIHC) research subject database. All pre-lingually deafened CI children received their CI(s) before 3.5 years of age with the Cochlear CI system. All post-lingually deafened adult participants had acquired hearing loss after the age of 20, i.e., after they developed typical speech and language skills.

All CI subjects had more than 1 year of CI experience at the time of testing. They had seen audiologists within 6 months of the testing session or saw audiologists on the same day of testing. All CI subjects were confirmed by their audiologists to be successful, full-time CI users, with their devices functioning appropriately at the time of the study, as verified through subject reports and listening checks. Parents and audiologists did not express any concerns about the development of the child; all children had not been diagnosed with other difficulties. Table 2 shows the age distribution of participants in the CI group. One 10-year-old participant was unable to complete testing in noise due to time constraints and was therefore excluded from further analysis. Stimulus artifacts from CI systems can hinder the ability to clearly observe neural potentials of interest (Hu et al., 2024a), particularly when the stimulus duration overlaps with the latency of the neural response, as seen in the ACC paradigm (Undurraga et al., 2021; Jeon et al., 2023, 2025). To address CI artifacts, our laboratory employs techniques such as band-pass filtering (1–30 Hz) and balancing electrode impedances. These strategies, along with others detailed in Jeon et al. (2025), are effective in reducing artifact interference. However, despite these measures, data from two participants (aged 13 and 20) were affected by artifact contamination and were excluded from further analysis.

**Table 2:** Age distribution of participants with cochlear implants.

	Pre-lingually deafened cochlear implant users																		Post-lingually deafened
Age (in years)	3	4	5	6	7	8	9	10	11	12	13	14	15	16	17	18	19	20–30	27–62
*n* = 59	2	1	3	2	4	3	4	2	1	4	4	1	4	3	1	4	0	5	11

### 2.2 General procedures

Both NH and CI listeners were tested using the same equipment and procedures to present stimuli and record CAEPs. Each subject sat in a comfortable chair in a sound booth. Speech stimuli consisting of two vowels were presented at 65 dBA via a loudspeaker located at 0-degree azimuth approximately 1 meter from the listener. All stimuli were presented once in quiet and once in noise conditions. Subjects were encouraged to read, play with an iPad, or watch captioned videos with no sounds to stay alert during testing. To minimize movement-related noise interference, participants were specifically instructed to use the iPad with minimal hand or body movement. They were informed that they would be monitored using two video cameras with an audio system mounted in the sound booth to ensure they were awake, comfortable, and minimizing unnecessary movement.

For recording, a total of seven disposable surface electrodes were used to create four channels for all NH and CI subjects. Three differential channels were allocated for recording CAEPs: Cz-to-right mastoid, Cz-to-left mastoid, and Cz-to-inion (Oz). One channel was used to monitor eye blinks, and the seventh electrode used as the ground. To minimize CI artifact, bilateral CI users were asked to wear only one CI during testing. The better hearing ear, according to the participant or parents’ report, was selected for testing. If the person had CIs sequentially, the one implanted before 3.5 years of age was selected. If there was no preferred test ear or if it was a simultaneous implantation, the right ear was selected, and the CI on the left side was removed. For CI users who used a HA in the contralateral ear, the HA was also removed to reduce potential artifact interference, and the ear was plugged.

This study required one visit of 2 h for all subjects, including preparation, recordings, and two short breaks. Additional breaks were provided to subjects as needed.

### 2.3 Electrophysiologic measures

#### 2.3.1 Stimuli

Two 800-ms-long stimuli, consisting of 400-ms-long quasi-synthetic vowels, were procedurally created. First, a male English speaker produced the vowels /u/ and /i/. These naturally spoken vowels were recorded at a sampling rate of 44,100 Hz in the WAV sound format. Adobe Audition 1.5 (Adobe^®^ Systems Inc., San Jose, United States) was used to modify the recorded waveforms. One cycle of each vowel was selected and repeated for longer than 400 ms by copying and pasting each vowel cycle at zero crossing. Using this method, two vowel segments (/u/ and /i/) with steady amplitude were created. Each vowel segment was truncated to 400 ms.

**Figure 1:**
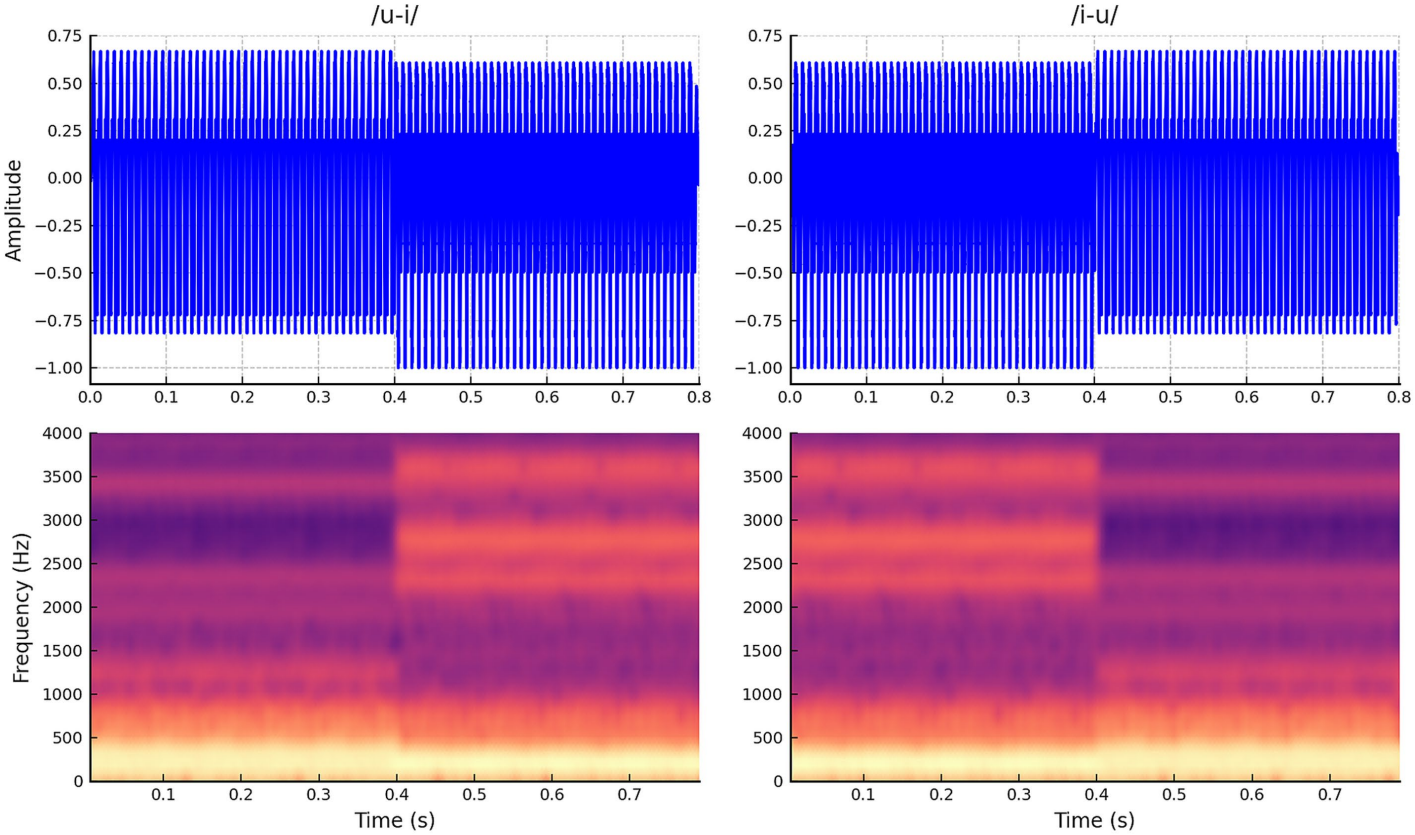
Waveforms and spectrograms for stimuli. This stimulus includes two vowel sounds that change halfway through (400 ms after onset) from /i/ to /u/ and vice versa from /u/ to /i/.

The formant frequencies of /u/ were F1 = 316 Hz, F2 = 1,178 Hz, and F3 = 2,306 Hz. The formant frequencies of /i/ were F1 = 254 Hz, F2 = 2,270 Hz, and F3 = 2,701 Hz. Prior to connecting the two quasi-synthetic vowels, /u/ and /i/ were adjusted to match the average RMS amplitudes. The two vowels were concatenated at the zero crossing, i.e., the last sampling point of one vowel was joined to the first sampling point of the following vowel. This process created two 800-ms-long stimuli: one beginning with the quasi-synthetic vowel /u/ followed by /i/ and the other beginning with /i/ followed by /u/. Creating stimuli in pairs (/u/−/i/ and /i/−/u/) allows us to measure both the onset P1-N1-P2 and the ACC responses for each segment of the stimulus. No discontinuity was observed in the waveforms, and spectral splatter was marginal in the spectra. No audible clicks or pops were present in the stimuli, /u/−/i/ and /i/−/u/. These two stimuli were tapered over 5 ms at the onset and the offset using fade-in and fade-out cosine functions equipped in Adobe Audition. The final two stimuli, /u/−/i/ and /i/−/u/, were again equalized in terms of overall RMS. Figure 1 shows the waveforms and spectra for the two 800-ms-long stimuli.

#### 2.3.2 Sound presentation: in quiet and noise conditions

A PC-based MATLAB program was used to read each 800-ms-long speech stimulus. This MATLAB program included a low-pass filter with a cutoff frequency of 8 kHz to attenuate any energy above this frequency in the speech stimuli. Additionally, the program added a 300-ms-long baseline prior to each stimulus and incorporated a time delay between stimulus presentations to ensure a slow presentation rate, averaging one presentation every 3.3 s. The program varied the length of the interstimulus interval (ISI), onset-to-onset of the stimulus, randomly between 2.8 and 3.8 s to minimize the effects of adaptation.

The stimuli, processed via the MATLAB program, were routed to the Grason-Stadler GSI-61 audiometer. The sound level was controlled by the GSI-61 to achieve 65 dBA at the subject’s ear level, with the subject positioned approximately 1 meter from the loudspeaker in a double-walled sound booth. All speech stimuli were presented once in quiet and once with background noise. In quiet conditions, each stimulus was presented alone at approximately 65 dBA through the loudspeaker located at 0° azimuth. In noise conditions, each stimulus was presented with background noise at 55 dBA, creating a + 10 dB signal-to-noise ratio (SNR), using the same loudspeaker. The background noise consisted of speech-weighted noise built into the audiometer, with equal energy per frequency from 0.25 to 1 kHz and a roll-off of12 dB per octave from 1 to 6 kHz. All stimuli and listening conditions were presented in a randomized order for all subjects. Each stimulus was presented in 200 trials per condition, achieved by combining four recordings of 50 sweeps per stimulus per condition. This resulted in a total of 400 sweeps across both listening conditions.

#### 2.3.3 Recording parameters

A total of seven surface electrodes were applied to the scalp and face, with impedances maintained below 5,000 ohms and differences between electrodes within 2000 ohms. Impedance levels were checked before and midway through the recordings. The electrode locations were as follows: vertex (Cz), right mastoid, left mastoid, inion, forehead, and above and below the eye. The vertex (Cz) electrode served as the active, non-inverted electrode (+), while the right and left mastoid, and inion electrodes served as references, i.e., inverted electrodes (−). Three differential recording channels were created to record the onset P1-N1-P2 and ACC responses. The first channel recorded activity between the vertex (Cz) and the right mastoid, the second channel between the vertex (Cz) and the left mastoid, and the third channel between the vertex (Cz) and inion (Oz). The purpose of using these channels was to explore potential differences; however, no significant differences were observed. As a result, the three-channel signals for each subject were averaged. An additional channel was created to monitor eye blinks using electrodes placed above and below the eye contralateral to the CI. Traces with potential contamination from eye blinks were rejected online. A ground electrode was placed on the forehead.

An optically isolated Intelligent Hearing System (IHS) 8-channel Opti-Amp (Model: IHS-3678) differential amplifier was used to record the ongoing EEG activity with a gain of 10,000 and a band-pass filter between 1 and 30 Hz. A National Instruments Data Acquisition board (DAQCard-6062E) was used to sample the ongoing EEG activity at a rate of 10,000 Hz. Custom-designed LabView software was utilized to display each waveform after each stimulus and to generate averaged responses online in all three auditory evoked potential recording channels. This software also allowed for monitoring waveforms from eye movements online. The recording time window was 1,400 ms, starting 300 ms prior to each 800-ms-long stimulus.

For each subject, a total of 400 accepted recordings were obtained for the two speech stimuli across both listening conditions. This was achieved by combining four separate recordings of 50 sweeps per stimulus in each listening condition. Each block of 50 trials required approximately 2.75 min to complete when the stimulus presentation (including the ISI) lasted 3.3 s. After every 50 sweeps, the listening condition and/or stimulus was switched randomly, allowing the subject a short break. Including these breaks, transition times, and any additional sweeps required due to artifacts, each block of 50 sweeps took approximately 4 to 5 min. The total recording and preparation time for each subject was slightly longer than 1 h.

### 2.4 Analysis

#### 2.4.1 Grand average waveforms

The obtained evoked potentials were analyzed offline using a custom MATLAB program. Each subject had a total of two average waveforms (400 sweeps each) for cortical auditory evoked potentials, recorded using speech stimuli (/u-i/ and /i-u/ combined) in both quiet and background noise listening conditions. For this study, recordings from the two stimuli were combined to facilitate data processing and analysis. While preliminary observations suggest no significant differences between the stimuli, further analysis is needed to confirm this. Grand average responses were calculated for subjects grouped by one-year and three-year age intervals to illustrate how the morphology of the onset P1-N1-P2 and ACC responses changes as a function of age.

#### 2.4.2 Peak latencies and N1-P2 peak-to-peak amplitudes

Latencies of identified peaks (P1, N1, P2) and peak-to-peak amplitudes of N1-P2 for both onset and ACC responses were analyzed using a customized peak-picking program in MATLAB. Means and standard deviations of peak latencies and amplitudes are reported.

The change in P1 latency with age is of particular interest for comparing developmental patterns of the onset and the ACC, as the P1 component is present in all age groups and is a known biological marker for central auditory system development from previous studies of the onset response. Linear regression was used to test the effect of age on P1 latency for both the onset and the ACC. A two-sample t-test was conducted to determine whether the slopes of the onset and ACC regression lines are significantly different from each other. Additionally, paired t-tests were performed as post-hoc analyses to compare P1 latency between the onset and the ACC, as well as between quiet and noise conditions.

Since the N1 peak is known to be absent in young children and starts to emerge later in life, the detectability (in percentage) of the N1 component and the N1-P2 peak-to-peak amplitude for the onset and the ACC were compared in each age group. Linear regression and a comparison of two slopes were also used to test the age effect on N1-P2 amplitudes and to determine if they differ between the onset and the ACC. Again, a paired t-test was used to compare N1-P2 amplitudes between the onset and the ACC and between quiet and noise conditions.

#### 2.4.3 Correlations between adults’ and children’s grand average waveforms

To quantify developmental changes, correlations between waveforms at each age group were calculated relative to NH adult responses. This assessment helps determine the similarity of waveforms at each age compared to normal adult responses, allowing for an easy comparison of developmental patterns between the onset and the ACC in each listening condition. Correlations between adults and children were calculated for the recordings within a 250 ms duration after stimulus onset and after a stimulus change. This 250 ms window was selected because both onset P1-N1-P2 and ACC responses occur within this latency in mature cortical auditory systems. To calculate correlations among adults, grand average waveforms from half of the adults were compared with grand average waveforms from the other half. This comparison was conducted to assess the noise level of correlations in adults and evaluate random variability across two subgroups. When correlation coefficients are plotted as a function of age, they quantitatively show changes in the morphology and amplitude of the onset P1-N1-P2 and ACC responses with increasing age.

#### 2.4.4 Statistics

All statistical analyses were conducted using SAS (Statistical Analysis System, version 9.3). The analyses were applied consistently for both quiet and noise listening conditions to maintain comparability of results across conditions. To assess the effect of background noise on developmental patterns of the onset and the ACC, responses were compared between quiet and noise conditions using latency, amplitude, and correlation data as described above. Analysis of variance (ANOVA) was used to compare onset and ACC responses among three adult groups (NH listeners, pre-lingually deafened CI users, and post-lingually deafened CI users) in both quiet and noise listening conditions. ANOVA was chosen because it allows for the comparison of multiple groups simultaneously while accounting for variability within each group. Post-hoc tests were conducted following significant ANOVA results to identify specific group differences.

## 3 Results

While the morphology of responses varied with age, both onset P1-N1-P2 and ACC responses were present in all waveforms obtained using long-duration speech-like stimuli from NH and CI subjects in both quiet and background noise conditions.

**Figure 2:**
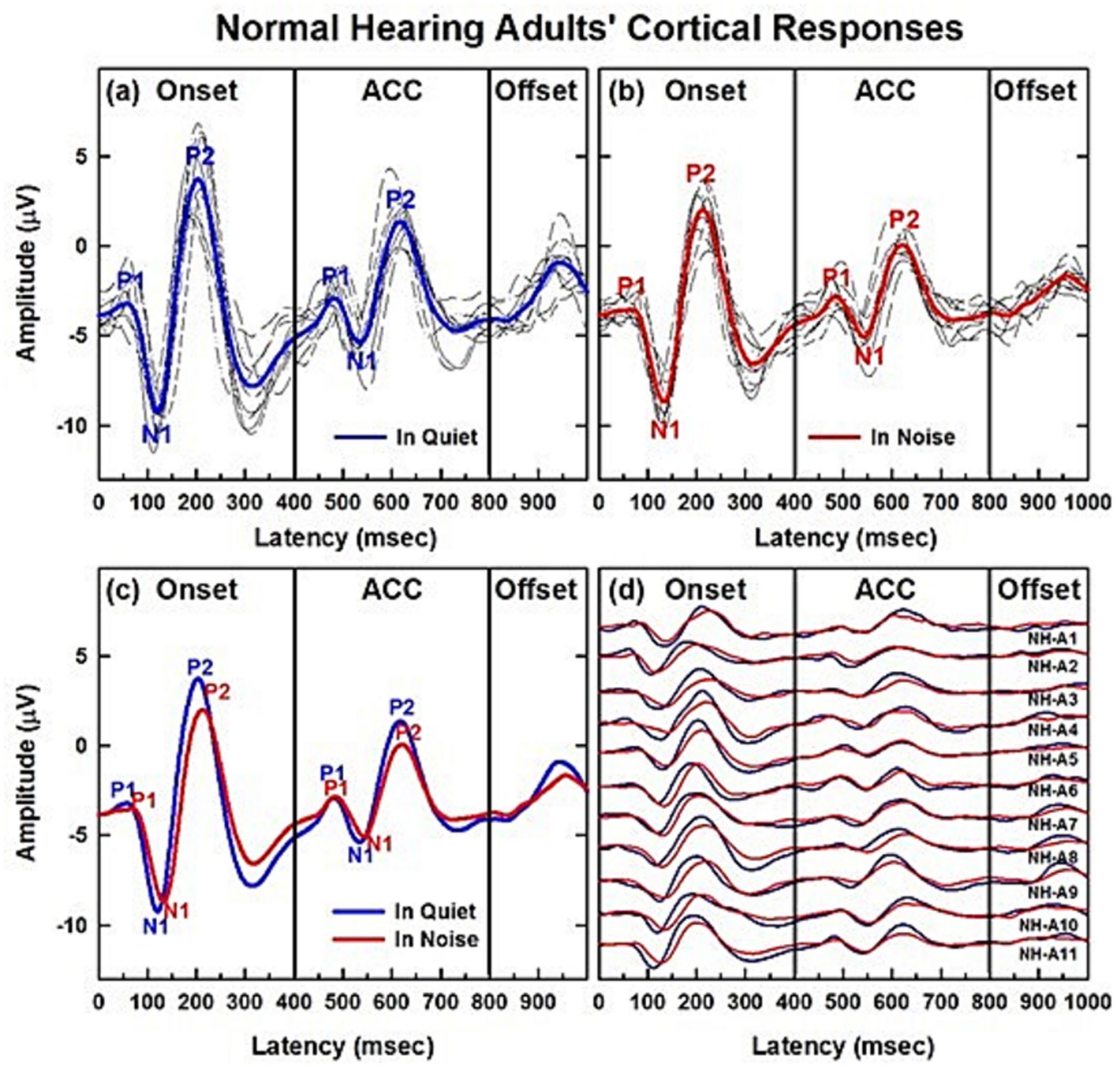
CAEPs of normal hearing adult listeners in quiet and noise conditions. **(a,b)** Show cortical responses obtained from eleven NH adults. **(a,b)** Show onset and ACC responses obtained in quiet and background noise (+10 dB SNR using speech-shaped noise) conditions, respectively. Individual data are shown in black and grand average waveforms in blue and red for each listening condition. **(c,d)** Compare the two listening conditions in grand average waveforms and individual subjects, respectively.

Before discussing the detailed results of NH and CI children, Figures 2a–d present these cortical responses in NH adults, which represent individuals with mature central auditory systems. Figures 2a–b show onset and ACC responses obtained in quiet and background noise conditions, respectively. Individual data are shown in black, and grand average waveforms from 11 NH adults are shown in blue for quiet and in red for background noise conditions. The variability across subjects was small in both listening conditions. Figure 2c compares grand average waveforms obtained from the two listening conditions, and Figure 2d shows the comparison of listening conditions for individual waveforms. Adding background noise (in this case, speech-shaped noise at 10 dB SNR) did not change the morphology for onset and ACC responses. However, it did significantly increase latencies and decrease N1-P2 peak-to-peak amplitudes for both onset and ACC responses at the *α* = 0.01 level (see Supplementary Table S1).

Details are provided in Supplementary Table S1, which summarizes the latencies and amplitudes of both onset and ACC responses across the two listening conditions. The table presents means and standard deviations of responses measured from NH adults (*n* = 11), along with results of paired t-tests comparing the two conditions. Additional results in the Supplementary materials describe how these responses are influenced by age and hearing loss in CI users.

### 3.1 Changes of CAEPs with age in NH listeners in quiet

**Figure 3:**
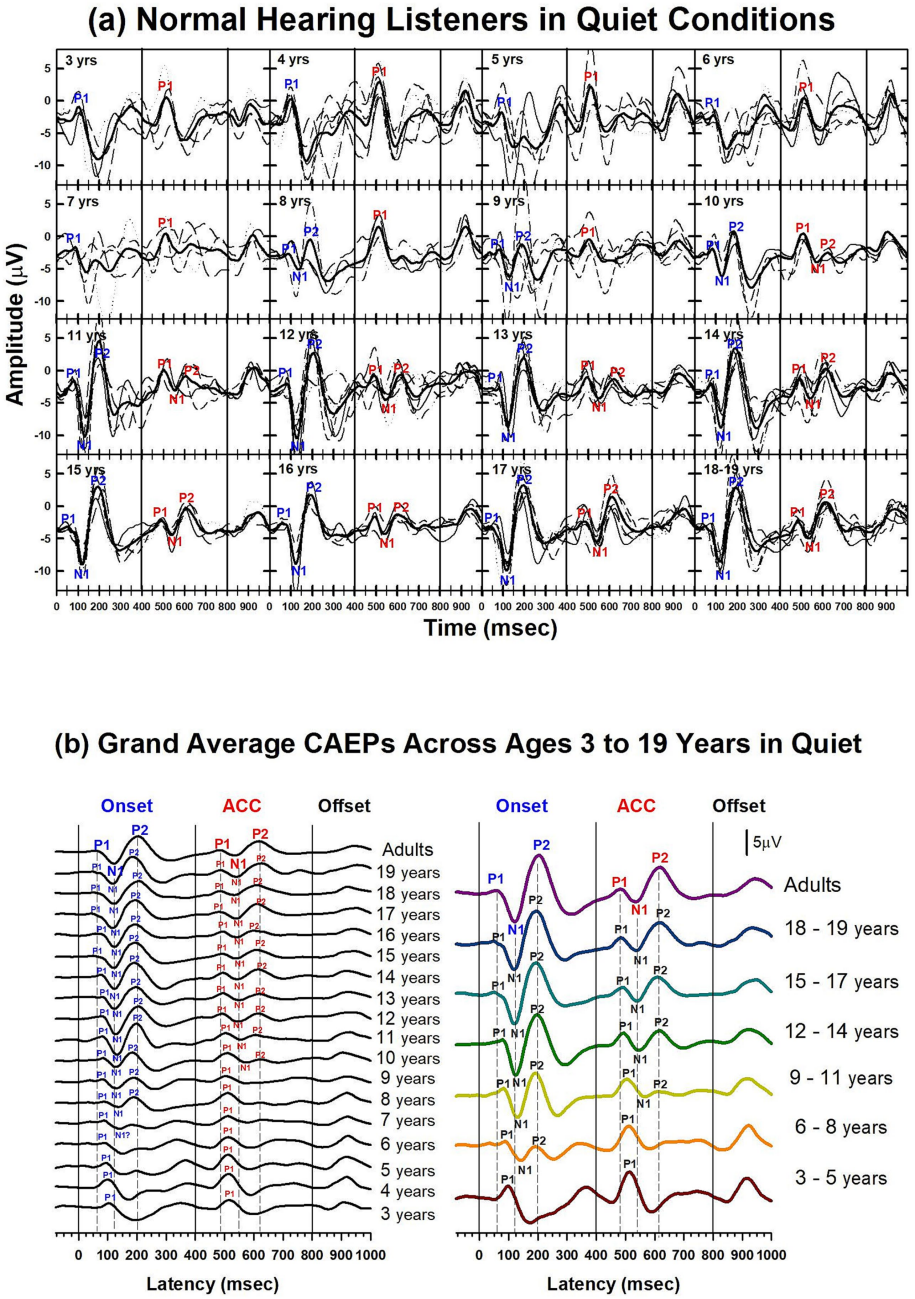
Waveforms of CAEPs in NH listeners in quiet. **(a)** Grand average CAEP waveforms recorded at electrode Cz are shown for individual age groups ranging from 3 to 19 years. Each subplot displays responses from both /u-i/ and /i-u/ stimuli combined. Thin lines represent individual recordings; thick black lines represent grand averages for each age group. Key waveform components (P1, N1, P2) are labeled at both the onset and acoustic change complex (ACC) time points. **(b)** Left panel: grand average CAEPs across ages 3 to 19 years and adults, showing developmental trends in response morphology over time. Right panel: age groups are collapsed into 3-year intervals to illustrate group-level developmental patterns. Onset, change, and offset of the stimulus occur at 0, 400, and 800 ms, respectively (marked by solid vertical lines). Dashed lines indicate the peak latencies of P1, N1, and P2 components in the adult group for both onset and ACC responses.

**Figure 4:**
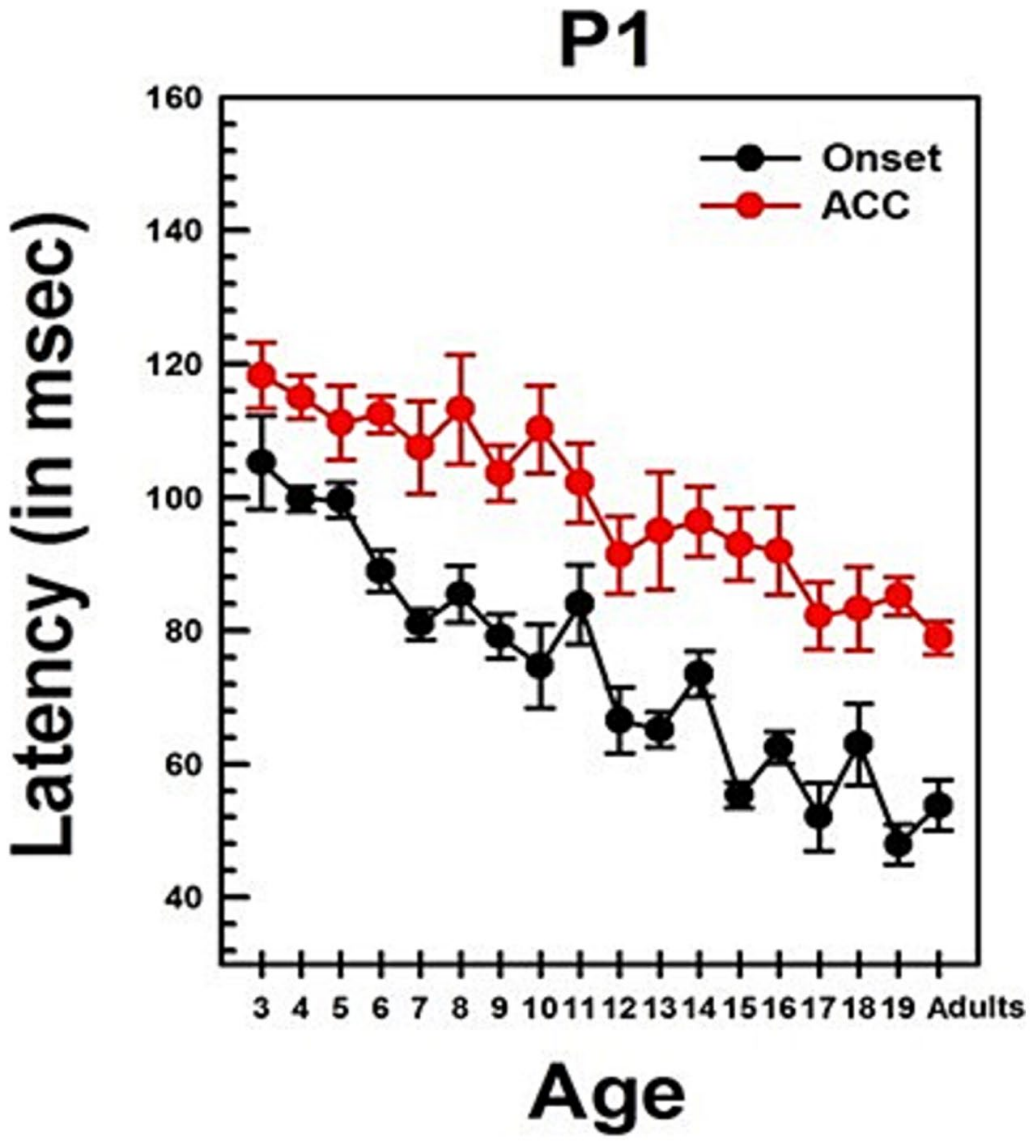
Changes in P1 latency with Age in NH listeners in quiet. This graph shows changes in P1 latencies for both onset and ACC responses. Black dots indicate data from the onset response, and red dots indicate data from the ACC. Error bars represent standard errors. The P1 latency of the ACC was calculated based on the post-stimulus change.

**Figure 5:**
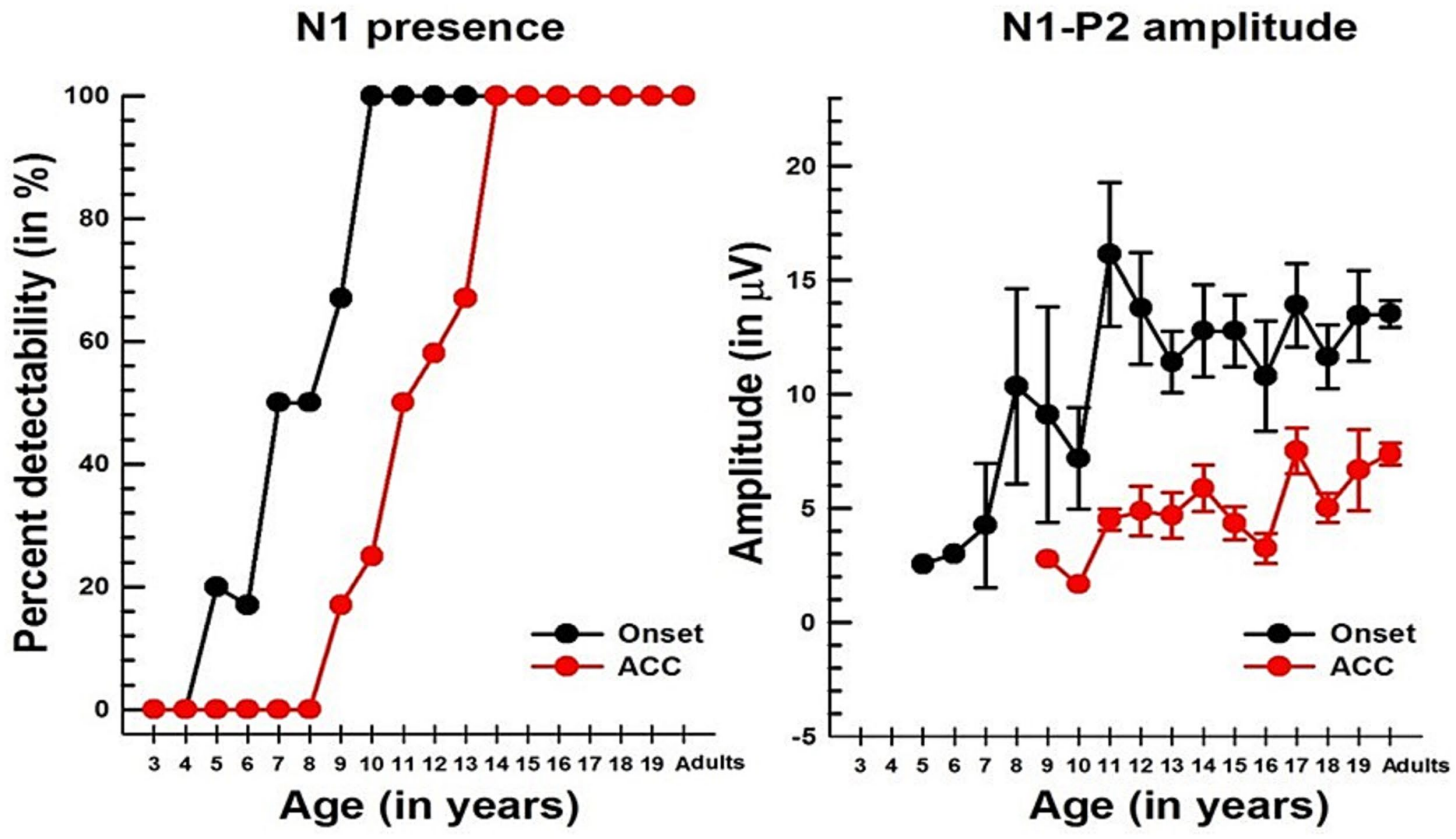
Changes in the N1 component with age in NH listeners in quiet. The left panel shows the detectability of N1, while the right panel shows the N1-P2 peak-to-peak amplitude with age for both onset and ACC responses. In both graphs, black dots indicate data from the onset, and red dots indicate data from the ACC. Error bars represent standard errors.

**Figure 6:**
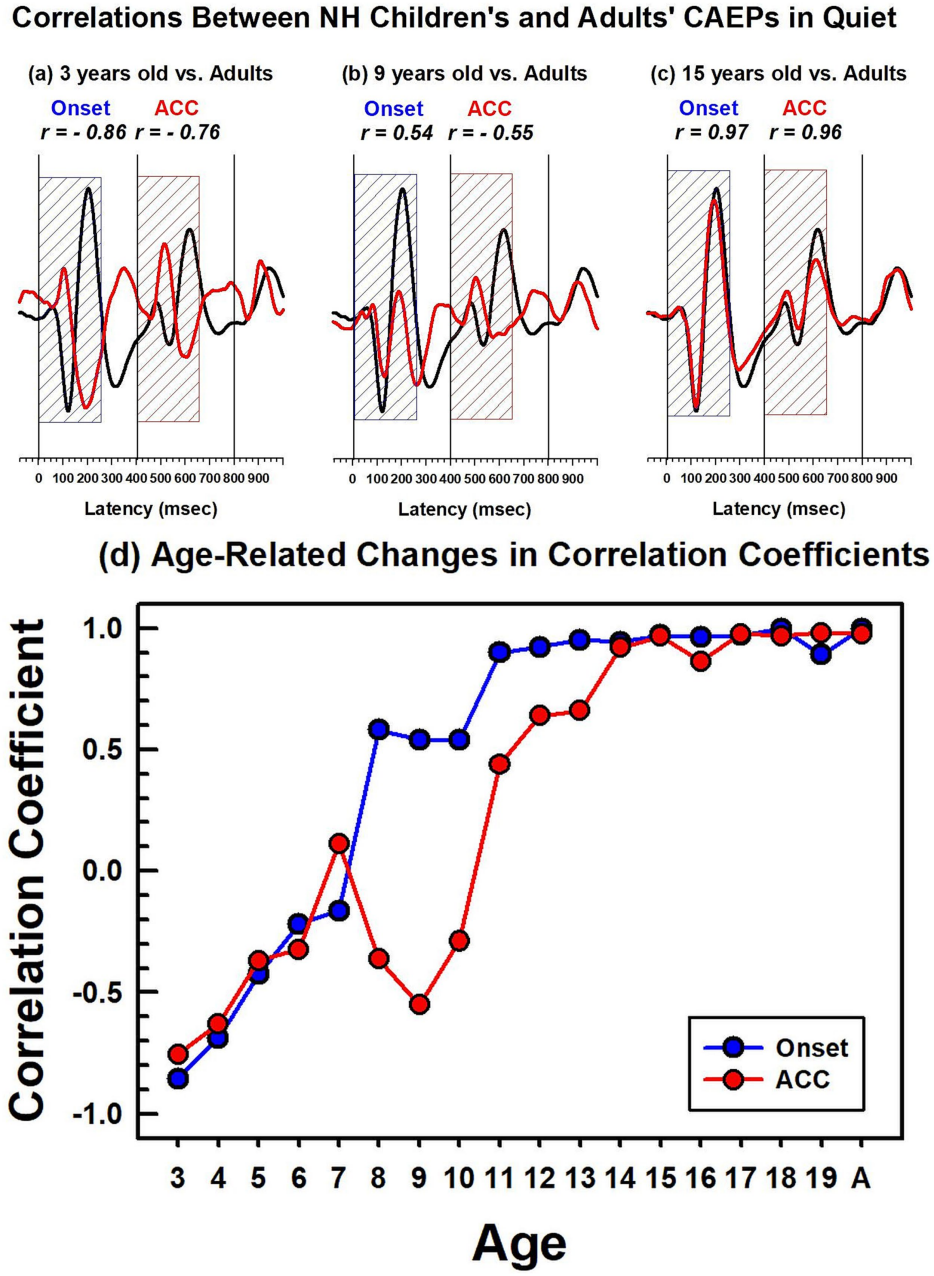
Correlations between NH children's and NH adults' CAEPs in quiet. The top panel figures **(a–c)** show examples of correlation coefficients between adults’ and children’s grand average waveforms obtained in quiet. The adults’ grand average waveform is shown in black solid lines, and the 3-, 9-, and 15-year-old’s grand average waveforms are in red. The dashed blue and red boxes indicate the duration (250 ms) used for correlation analyses for the onset and the ACC, respectively. The bottom figure **(d)** shows calculated correlation coefficients for the onset, represented by a blue line with dots, and the ACC, represented by a red line with dots. For the adults’ noise level, the adult group was divided into two groups, and a correlation coefficient was calculated between the two adult groups.

Results of child NH listeners’ CAEPs recorded in quiet are shown in Figures 3–6. Figure 3 illustrates the morphological changes of the P1-N1-P2 and the ACC with age, recorded at electrode Cz. The top panel shows individual waveforms with dashed lines and the grand average waveform with solid lines for each age group.

To facilitate comparison across ages, the bottom left panel shows grand average waveforms for each age group, from 3 to 19 years old, and adults. P1 is present in all age groups for both onset and ACC responses. The N1 of the onset response emerges around 7 or 8 years old. The morphology of the onset P1-N1-P2 becomes adult-like at around 11 years old, with dominant N1-P2 components. The N1 of the ACC emerges later, at around 10 or 11 years old. The morphology of the ACC becomes adult-like later than the onset response, with the P2 amplitude becoming larger than P1 at 14 years old.

The bottom right panel shows grand average waveforms grouped in 3-year intervals for six groups (ages 3 to 5, 6 to 8, 9 to 11, 12 to 14, 15 to 17, and 18 to 19) and one adult group. For the onset P1-N1-P2, at 3 to 5 years, P1 is the dominant component with longer latencies than any other age group. At 6 to 8 years, the N1 peak begins to emerge in the onset response. At 9 to 11 years, the N1 and P2 responses become dominant, and the N1-P2 peak-to-peak amplitudes grow larger in older teenage groups. For ACC responses, P1 is the only component with a large amplitude from 3 to 8 years old. At 9 to 11 years, N1 and P2 peaks emerge, but P1 is still dominant. At 12 to 14 years, the N1 and P2 are dominant in the ACC, and the N1-P2 peak-to-peak amplitude grows in late adolescence.

Summary statistics of peak latencies and N1–P2 amplitudes for CAEPs recorded from all NH listeners in quiet conditions are provided in Supplementary Table S2. Traditionally, P1 latency has been used to characterize developmental changes in the onset response since it is observed in all age groups. Figure 4 illustrates the change in P1 latency with age for both the ACC and the onset response. At age 3, P1 latency was measured at 105 ms for the onset response and 118 ms for the ACC. In adults, it was measured at 54 ms for the onset response and 79 ms for the ACC.

Linear regression analyses show that the P1 latency for both the onset (*p* < 0.0001) and ACC (*p* < 0.0001) decreases significantly with age. For the onset response, the P1 latency shows a general decrease of approximately 3 ms/year on average, though the rate of change varies across different age groups. For the ACC, the P1 latency decreases by about 2 ms/year on average, with variability depending on the age range. Age alone predicts about 60% of the P1 latency variations in the onset response and 58% of the P1 latency variations in the ACC. Supplementary Table S3 shows the results of linear regression analyses on P1 latency with age and N1-P2 peak-to-peak amplitude with age for both onset and ACC responses. Data used for these analyses were collected from NH listeners in quiet listening conditions.

The slope of the onset P1 latency with age was significantly different from the slope of the ACC P1 latency with age, as determined by a two-sample t-test (t = 2.93, df = 168, *p* = 0.0038). This indicates that the developmental pattern of P1 latency may differ between the onset and the ACC. Additionally, P1 latencies of the onset and ACC responses were compared within subjects using a paired t-test. The P1 latency of the onset response was significantly shorter than the P1 latency of the ACC response (Mean = −24.90 ms, SD = 14, t = −16.99, df = 85, *p* < 0.0001).

Results of linear regression analyses examining the relationship between age and both P1 latency and N1–P2 amplitude in quiet conditions for NH listeners are provided in Supplementary Table S3.

Unlike P1, N1 was not observed in young children for either onset or ACC responses, but it emerged in older children. Figure 5 shows the course of development in the detectability of N1 (left panel) and the peak-to-peak amplitude of the N1 and P2 (right panel). For the onset response, N1 was detectable at 5 to 6 years in about 20% of subjects, reached about 50% at 7 to 8 years old, and was present in all subjects over the age of 10. For the ACC, the pattern is similar to that of the onset response; however, it does not emerge until 9–10 years old and is not present in all subjects until 14 years old.

Linear regression analyses show that the N1-P2 peak-to-peak amplitude increases significantly with age for both onset (*p* = 0.0222) and ACC responses (*p* = 0.0211). The slope of the N1-P2 amplitude is about 0.4 μV/year for the onset response and 0.3 μV/year for the ACC response. However, age predicts only about 10% of the variability in the amplitude of both onset and ACC responses. The slopes of regression for the onset and ACC N1-P2 amplitudes are not significantly different at the *α* = 0.05 level. Additionally, a paired t-test showed that the onset N1-P2 amplitude was significantly larger than the ACC N1-P2 amplitude across all age groups combined (Mean = 7.69 μV, SD = 4.10, t = 13.78, df = 53, *p* < 0.0001).

A comparison of CAEPs measured from children and adults is complicated because they have different morphologies, peak latencies, and amplitudes (Figures 3–5). To compare responses efficiently, a correlation analysis was conducted between the adults’ grand average waveform and the group grand average waveform for each age group. Since the onset and ACC occur during the first 250 ms after a stimulus onset and a stimulus change, data from this duration were used to calculate the correlation coefficient between adults and each age group.

Figures 6a–c show waveforms and calculated correlation coefficients for example age groups: 3 years, 9 years, and 15 years (shown in red) compared with the adults’ grand average waveform (shown in black). At 3 years, onset and ACC responses have peaks in opposite directions from the adults’ grand average waveform. They have strong negative correlations for both onset (r = −0.86) and ACC responses (r = −0.76). At 9 years, the onset response has a moderate positive correlation with adult responses (r = 0.54), but the change response has a moderate negative correlation (r = −0.54). This is when the onset morphology becomes more adult-like while the P1 is still a dominant peak in the ACC. At 15 years, both onset and ACC responses have strong positive correlations with adults’ responses (r = 0.97 and 0.96, respectively). Panel (d) summarizes calculated correlation coefficients for all age groups, with onset responses shown as blue dots and ACC responses shown as red dots. The correlation coefficient for onset responses plateaus around 11 years of age, while the ACC response correlation coefficient continues to increase and does not reach an adult-like plateau until approximately 14 years of age.

### 3.2 Changes of CAEPs with age in NH listeners in noise

**Figure 7:**
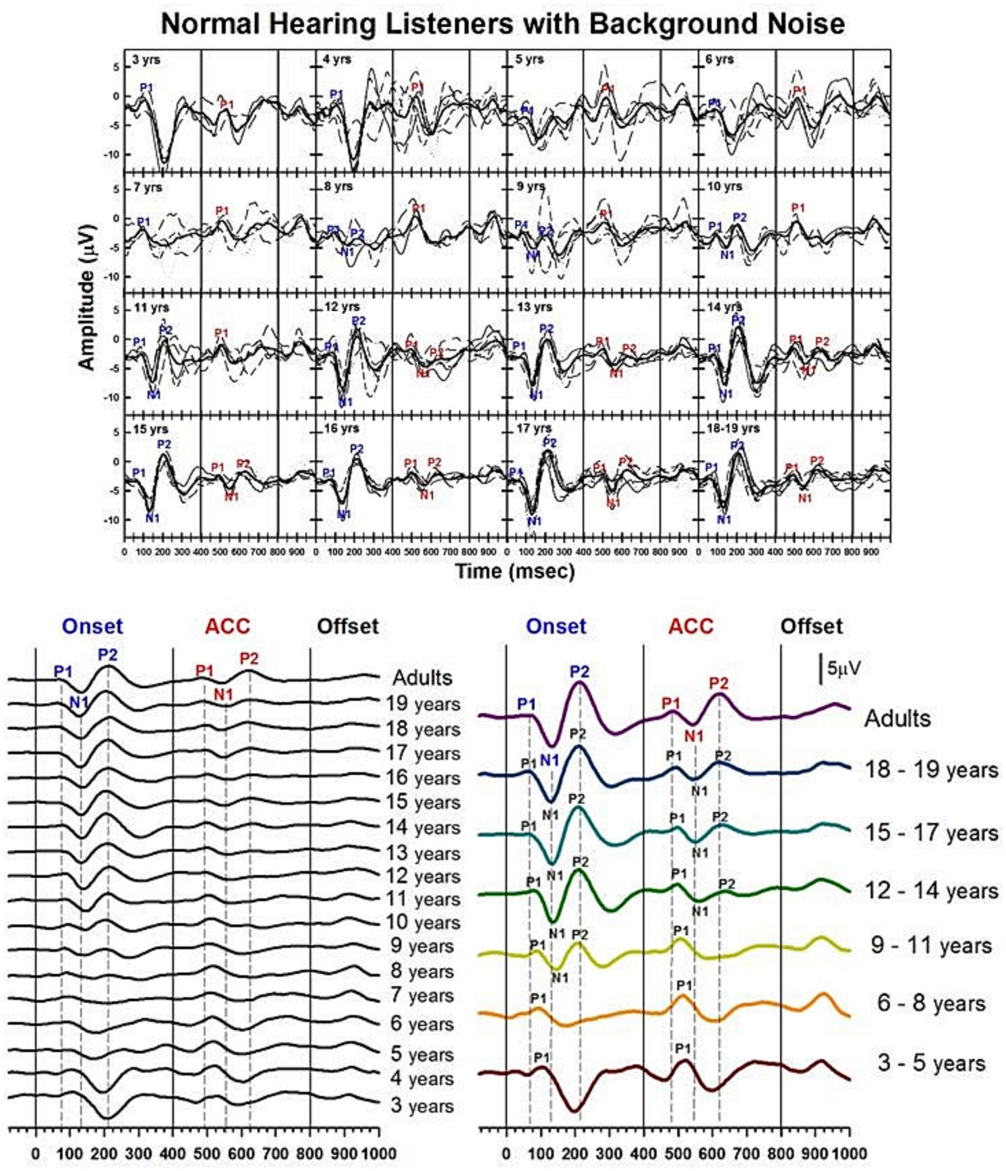
Waveforms of CAEPs in NH listeners in noise. Grand average waveforms are shown for NH listeners in background noise. The top panel displays individual waveforms and grand average waveforms for each age group. The bottom left panel presents a series of grand average waveforms from ages 3 to 19 years, and adults. The bottom right panel shows grand average waveforms for five groups of children, grouped in three-year increments, and adults. Three straight lines indicate when the onset, change, and offset of the stimulus occur. Dashed lines indicate when P1, N1, and P2 components appear in adults’ responses.

**Figure 8:**
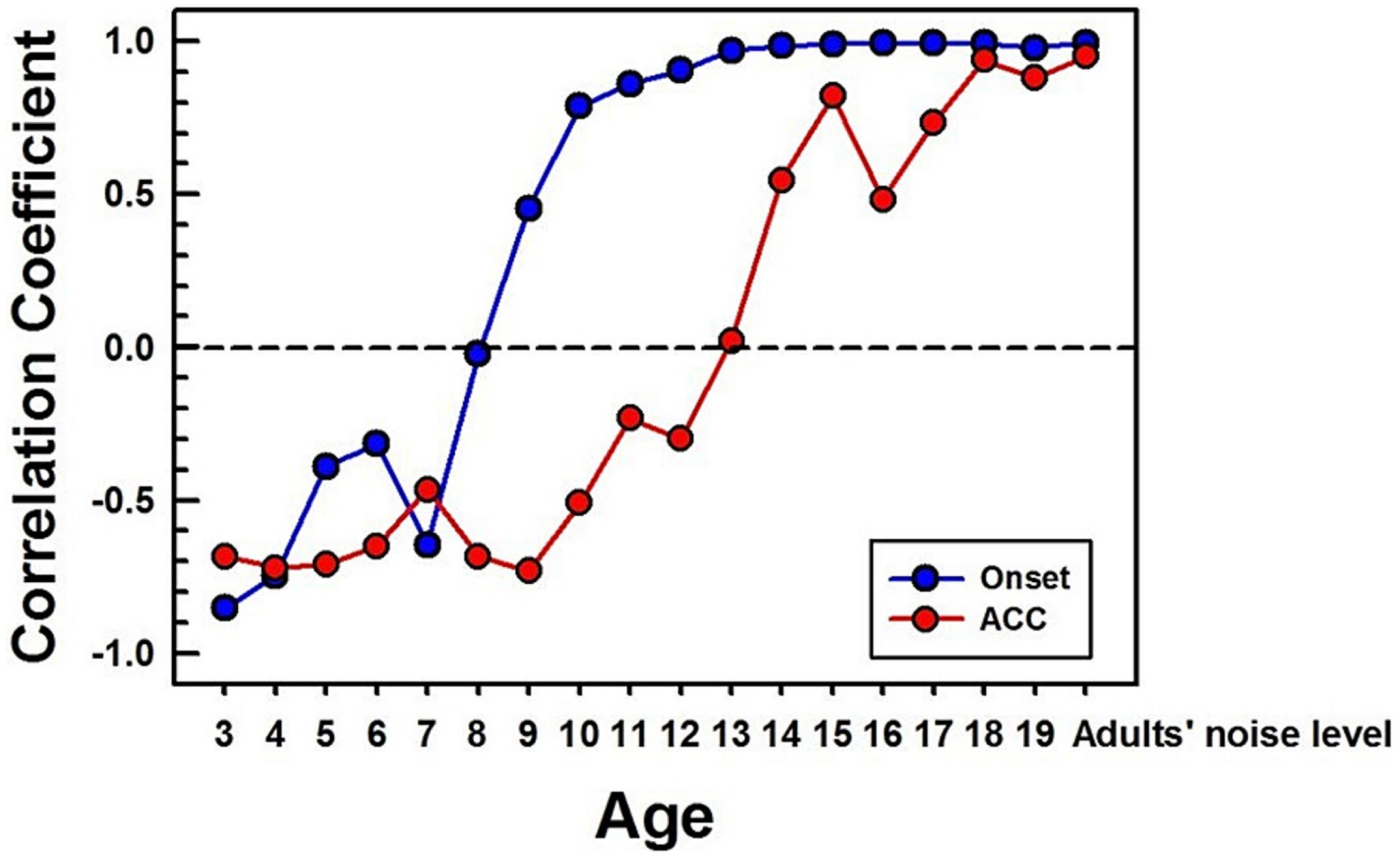
Correlations between NH children's and NH adults' CAEPs in noise. Correlation coefficients are calculated between each age group’s grand average waveform obtained with background noise and the adult’s in noise. The blue line with dots represents the onset response, and the red line with dots represents the ACC. The same time window (250 ms) after a stimulus onset and change was used for correlation calculation for onset and ACC responses, respectively. For the adults’ noise level, the adult group was divided into two groups, and a correlation coefficient was calculated between the two adult groups.

The secondary aim of the current study was to investigate how challenging listening conditions impact the developmental patterns of the onset and the ACC. Reliable CAEPs were measured with background noise for all subjects. Figures 7, 8 show CAEPs recorded from all NH listeners with speech-shaped noise at 10 dB SNR.

Figure 7 shows the morphological changes of the P1-N1-P2 and the ACC with age, recorded at electrode Cz with background noise. This graph is plotted in the same way as Figure 3, which showed CAEPs recorded in quiet conditions. The bottom right panel shows grand average waveforms from the three-year interval age groups. In onset responses, at 3 to 5 years, P1 is the dominant component with larger amplitude. At 6 to 8 years, while the amplitude becomes smaller, P1 is still the only clear component. At 9 to 11 years, the N1 and P2 peaks are evident in the onset response. The left panel shows that N1 may emerge as early as 8 years. At 12 to 14 years, the N1 and P2 responses become dominant in the onset response, and the morphology of the onset response becomes adult-like. For ACC responses, P1 is the only clear component between 3 and 11 years. N1-P2 peaks become evident at ages 12 to 14. The left panel shows that N1 becomes clear at around 12 years old. At ages 15 to 17 years, the N1-P2 response becomes larger in amplitude. The left panel shows that the morphology of the ACC becomes adult-like as early as 15 years, with P2 amplitudes larger than P1.

Summary statistics of peak latencies and N1–P2 amplitudes for CAEPs recorded from NH listeners in background noise (speech-shaped noise at 10 dB SNR) are provided in Supplementary Table S4.

Figure 8 shows calculated correlation coefficients for both onset (shown in blue) and ACC (shown in red) responses, using data obtained in noise. The correlation coefficients for the onset response plateau around 13 years old. However, the correlation coefficient for the ACC response continues to improve even into the late teenage years, reaching adult levels around 18 years old.

### 3.3 Changes of CAEPs with age in CI users in quiet

Previous sections have shown results of NH listeners in quiet and with background noise. The following sections will focus on how the onset P1-N1-P2 and the ACC change with age in individuals who were born deaf but received a CI early in life.

**Figure 9:**
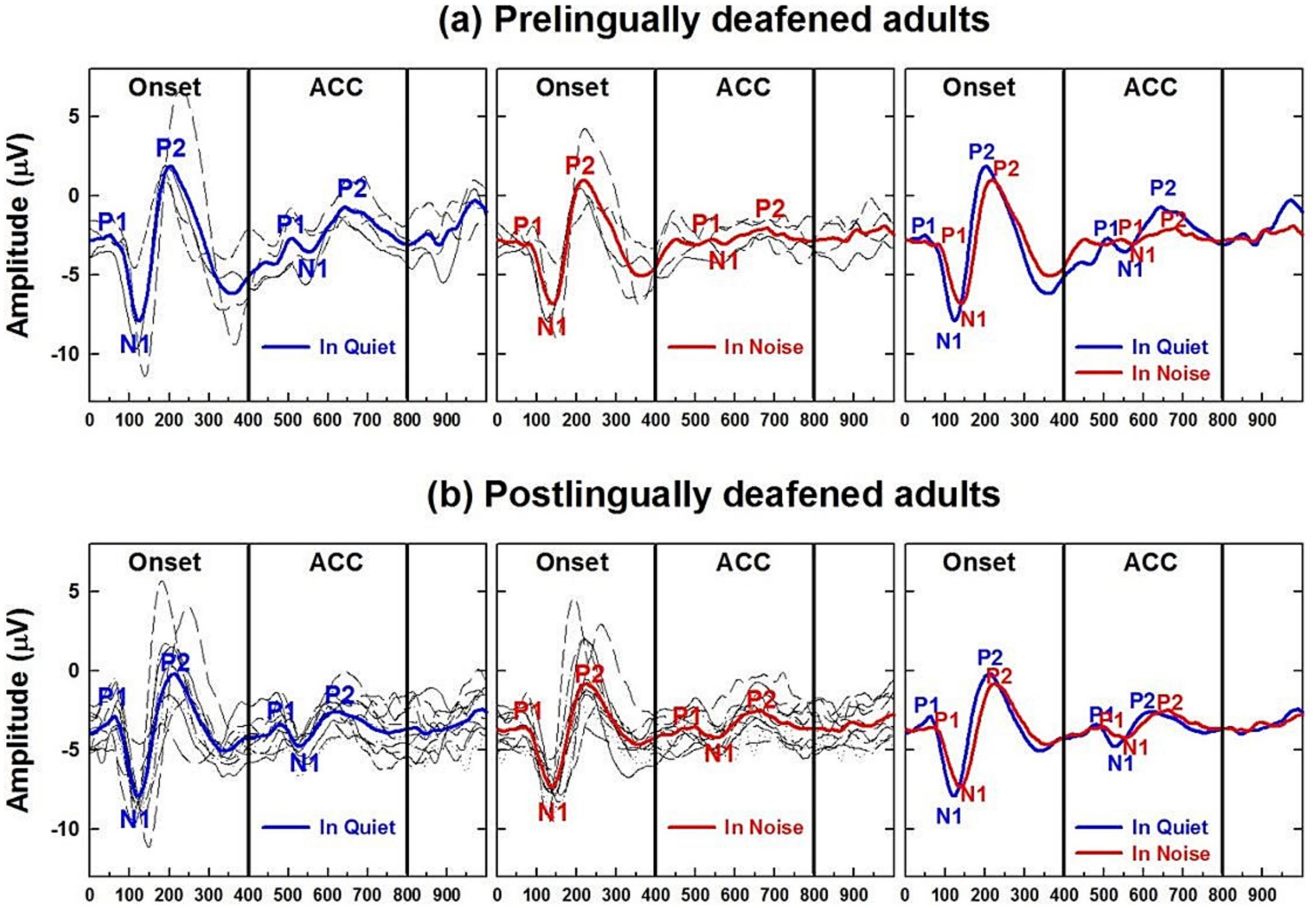
Adult CI users’ CAEPs in quiet and noise conditions. **(a)** Plots cortical auditory evoked potentials obtained from pre-lingually deafened CI adults and **(b)** from post-lingually deafened adults. The left and center panels show responses measured in quiet and noise listening conditions, respectively. Individual data are shown in black, and grand average waveforms are shown in blue and red for each listening condition. The right panel compares grand average waveforms between the two listening conditions for each of the CI groups.

Before examining the CI children’s data, Figures 9a,b present CAEPs measured in early-implanted, pre-lingually deafened adults, i.e., those who grew up with their CI, and post-lingually deafened adults, who received their CI after the age of 20. Onset and ACC responses obtained in quiet (the left panel) and with background noise (the right panel) are plotted for pre-lingually deafened (Figure 9a) and post-lingually deafened CI adults (Figure 9b). Individual data are shown in black, and grand average waveforms are shown in blue and red for the quiet and noise listening conditions, respectively. Similar to the results of NH listeners, adding 10 dB SNR background noise significantly increased peak latencies and decreased N1-P2 peak-to-peak amplitudes for both onset and ACC responses at the *α* = 0.01 level.

**Figure 10:**
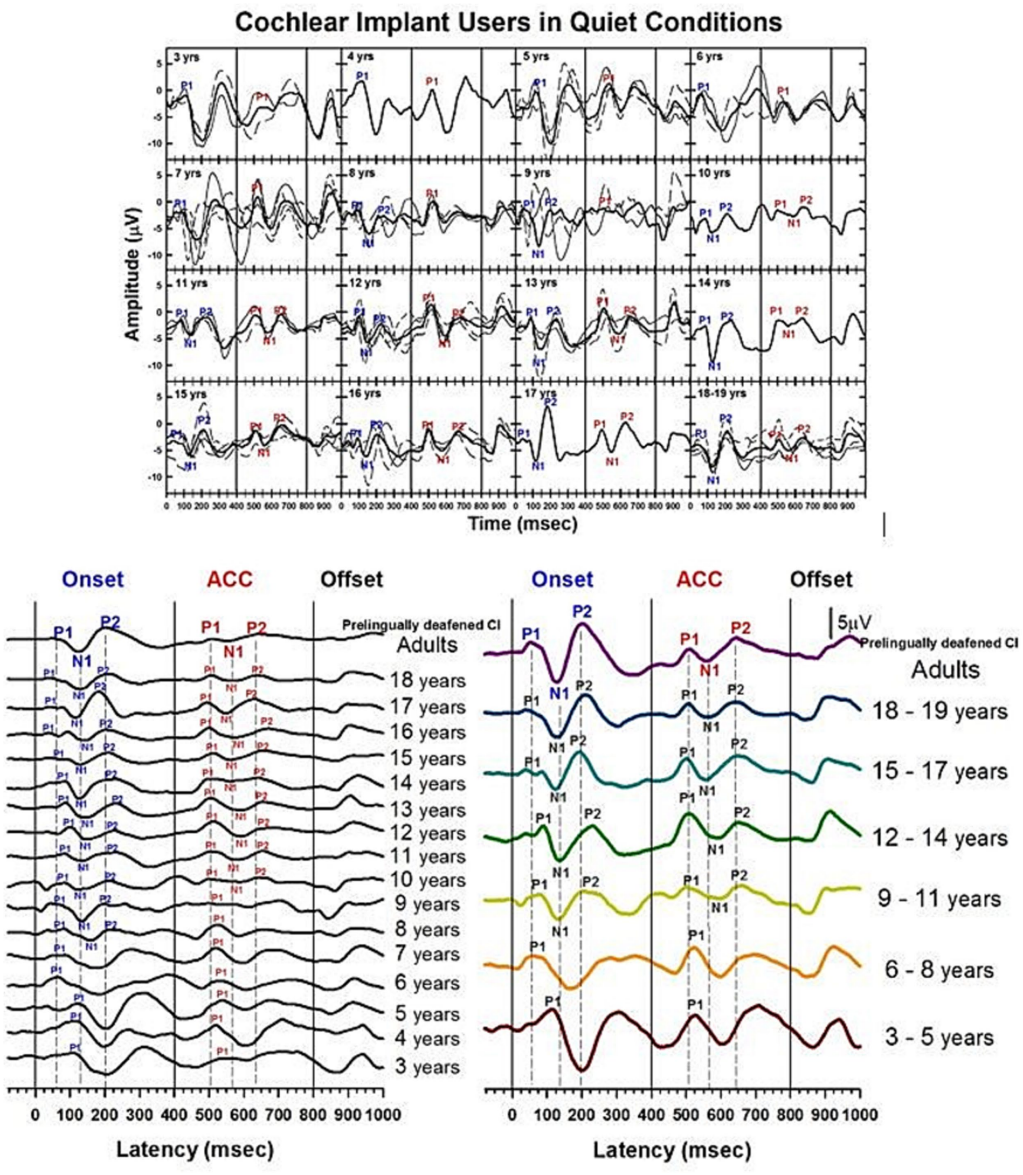
Waveforms of CAEPs in pre-lingually deafened CI users in quiet. Grand average waveforms measured at the electrode Cz are shown for early-implanted, pre-lingually deafened CI listeners. The top panel shows individual waveforms and grand average waveforms for each age group. The bottom left panel shows grand average waveforms for each age group ranging from 3 to 18 years, and adults. The bottom right panel shows grand average waveforms for five groups of children, grouped in three-year intervals, and adults. The three straight lines at 0, 400, and 800 ms indicate the onset, change, and offset of the stimulus, respectively. Dashed lines indicate when P1, N1, and P2 components are shown in adults’ onset and ACC responses.

**Figure 11:**
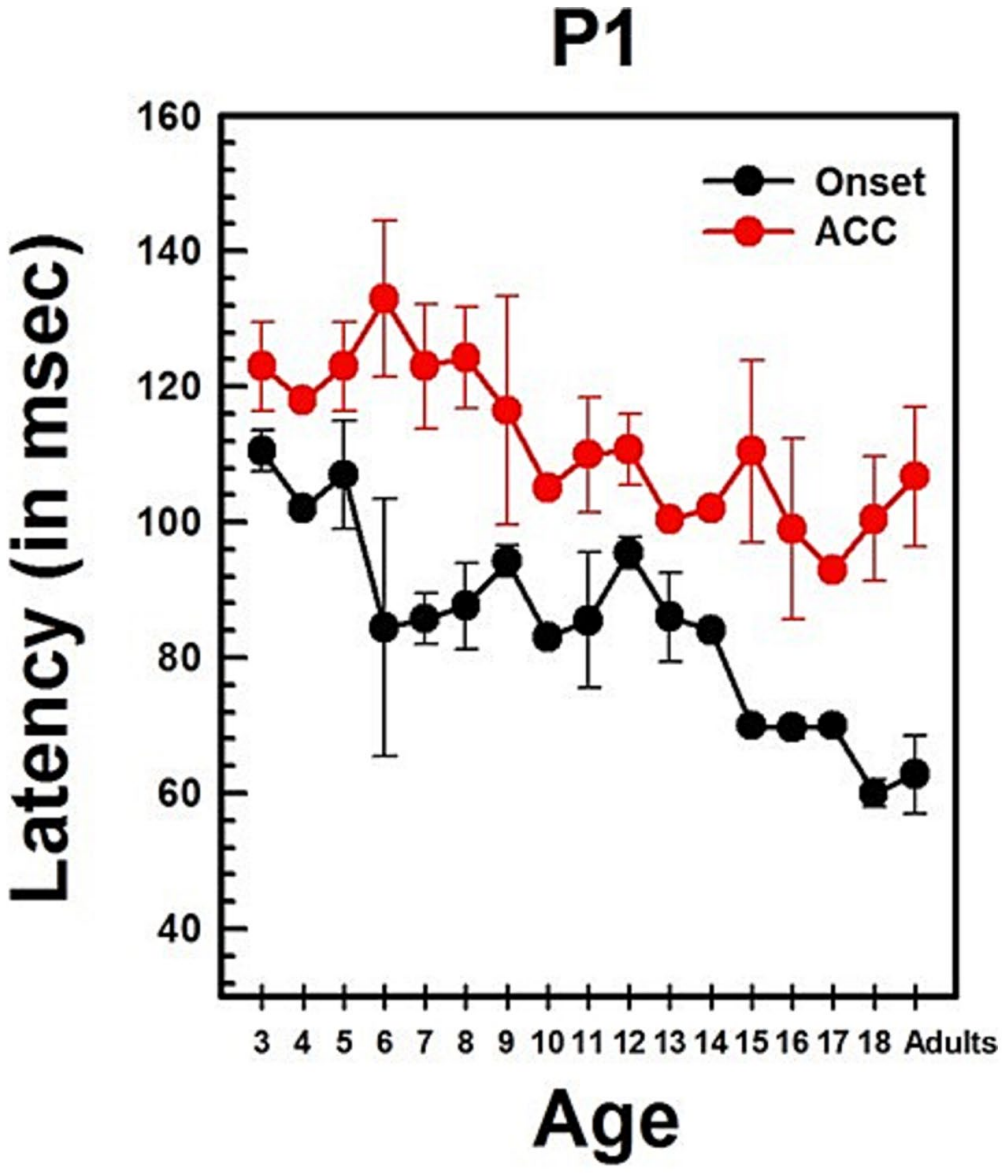
Changes in P1 latency with age in CI users in quiet. This graph shows changes in P1 latencies for both onset and ACC responses. Black dots indicate data from the onset response, and red dots indicate data from the ACC. Error bars represent standard errors. The P1 latency of the ACC was calculated based on the post-stimulus change.

**Figure 12:**
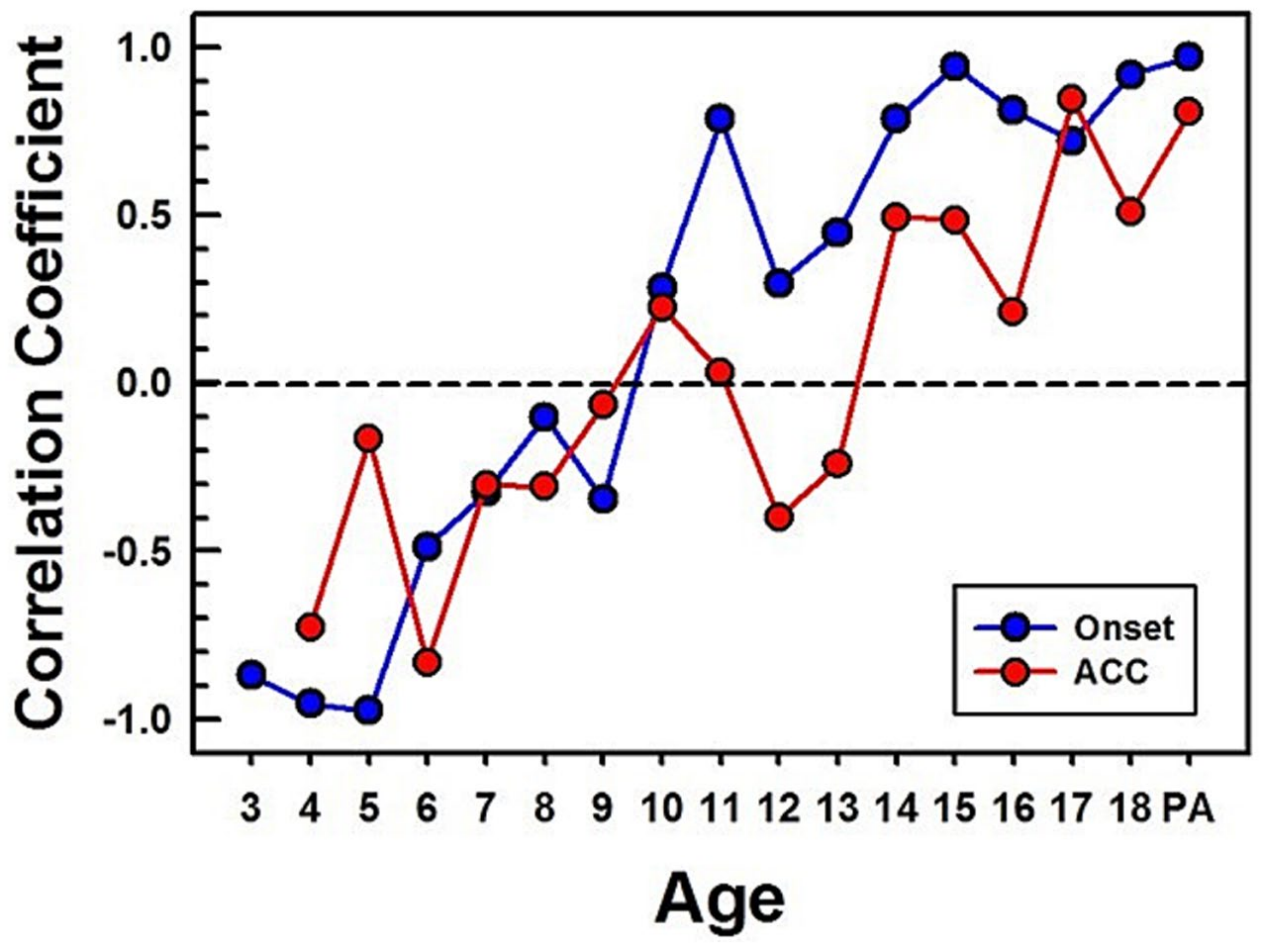
Correlations between CI children's and NH adults’ CAEPs in quiet. Correlation coefficients are calculated between CI children's grand average waveforms and NH adults' waveforms obtained in quiet. The blue line with dots represents calculated correlation coefficients for the onset, and the red line with dots represents the ACC. PA stands for pre-lingually deafened CI adults.

Developmental data from pre-lingually deafened CI listeners in quiet are shown in Figures 10–12. These plots parallel those presented for NH subjects. Figure 10 shows morphological changes of the P1-N1-P2 and the ACC with age. For onset P1-N1-P2 responses, P1 is the dominant component at 3 to 5 years, with longer latencies than any other age group. At 6 to 8 years, P1 is still present alone. The N1 of the ACC is evident as early as 8 years old in the grand average waveform (left panel). At 9 to 11 years, the N1 and P2 responses become dominant in the onset response, and their amplitude grows from ages 12–14. By ages 15–18, the morphology of the onset response becomes adult-like.

For ACC responses, P1 is the only evident component from 3 to 8 years of age. The N1 of the ACC appears as early as 10 years old in the grand average waveform (left panel). N1-P2 peaks are evident at 9 to 11 years, and their amplitude grows with age. The morphology of the ACC becomes adult-like at 15 to 17 years, with the P2 amplitude becoming larger than P1.

Summary statistics of peak latencies and N1–P2 amplitudes for CAEPs recorded from early-implanted, pre-lingually deafened CI users in quiet conditions are provided in Supplementary Table S6.

Previous studies show that early-implanted, pre-lingually deafened children exhibit similar P1 latency changes to those seen in NH children. Figure 11 illustrates the change of P1 latency with age for the ACC as well as for the onset in early-implanted, pre-lingually deafened children. This figure parallels Figure 4, which presents data for NH children.

Linear regression analyses show that the P1 latency for both the onset (*p* < 0.0001) and the ACC (*p* < 0.0001) decreases significantly with age. The P1 latency decreases by about 3 ms/year for the onset response and about 2 ms/year for the ACC. Age alone predicts about 35% of P1 latency variations in the onset and 51% of P1 latency variations in the ACC. The slope of the onset P1 latency with age was compared with the slope of the ACC P1 latency with age using a two-sample t-test. The two slopes are not significantly different (t = 1.25, df = 80, *p* = 0.214). This indicates that the developmental pattern of P1 latency may be the same for the onset and ACC responses. P1 latencies of the onset and ACC responses were compared within subjects using a paired t-test. The P1 latency of the onset response was significantly shorter than the P1 latency of the ACC response (Mean = −28.33 ms, SD = 20, t = −9.36, df = 41, *p* < 0.0001).

Table 3 presents the results of linear regression analyses examining the relationship between age and both P1 latency and N1–P2 peak-to-peak amplitude for onset and ACC responses in quiet conditions. These analyses were conducted using data from pre-lingually deafened CI users. The findings reveal significant age-related changes, particularly in the ACC responses, indicating ongoing cortical maturation in this population. Table 3. Linear Regression of age on P1 latency and N1–P2 amplitude in quiet for pre-lingually deafened CI users.

**Table 3:** Linear regression on P1 Latency and N1-P2 amplitude with age in quiet for pre-lingually deafened CI users.

	P1 latency - onset	P1 latency - ACC	N1-P2 Amplitude - onset	N1-P2 Amplitude - ACC
Slope	−2.795	−1.949	0.604	0.039
Intercept	115	134	−2.15	2.78
R-square	0.349	0.510	0.262	0.0056
*F* value	21.41	41.63	9.59	0.13
*P*	< 0.0001***	< 0.0001***	0.0045**	0.7216

* indicates *p* < 0.05, ** indicates *p* < 0.01, *** indicates *p* < 0.001.

Figure 12 shows calculated correlation coefficients between grand average waveforms of the early-implanted CI children and NH adults. The same method used for calculating correlation coefficients for NH (shown in Figure 6) was applied to CI children. All calculated correlation coefficients for the onset are shown in blue dots, and for ACC, in red dots.

The pattern of onset P1-N1-P2 development with age is noisy but similar to the normal hearing data. While the onset data from NH children plateau around 11 years old, the onset data from CI children reach high correlation coefficients at 11 years and plateau around 14 years old. The pattern of the ACC is also noisy but continues to improve with age. However, its correlation coefficient is lower than that of the onset in teenagers. Additionally, pre-lingually deafened CI adults (shown as “PA” on the x-axis in Figure 12) show higher correlation for the onset than the ACC with NH adults.

### 3.4 Changes of CAEPs with age in CI users in noise

**Figure 13:**
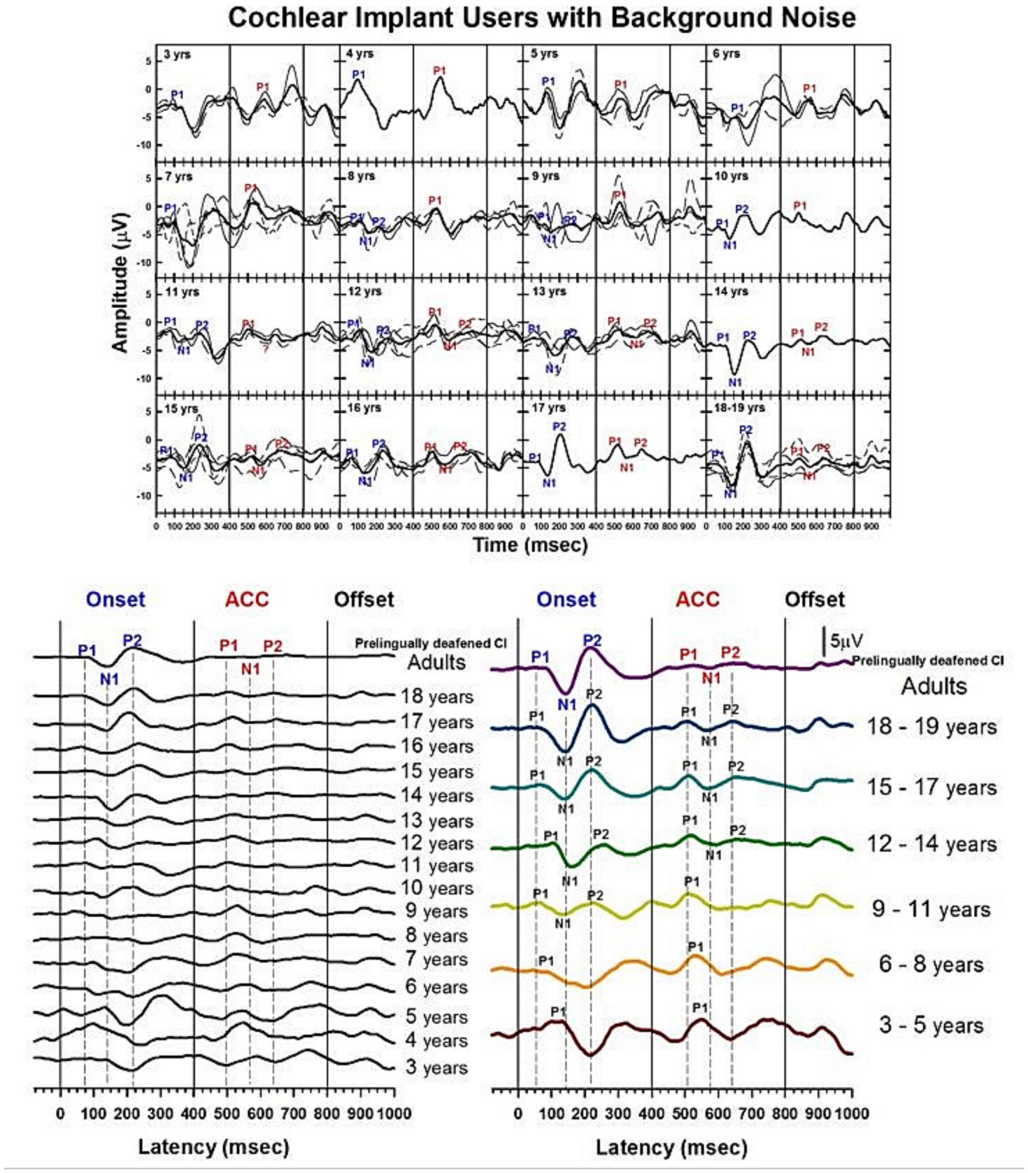
Waveforms of CAEPs in CI users in noise. Grand average waveforms are shown for early-implanted, pre-lingually deafened CI users in background noise. The top panel shows individual waveforms and grand average waveforms for each age group. The bottom left panel shows a series of grand average waveforms from ages 3 to 19 years, and adults. The bottom right panel shows grand average waveforms for five groups of children, grouped in 3-year increments, and adults. The three straight lines indicate when the onset, change, and offset of the stimulus occur. Dashed lines indicate when P1, N1, and P2 components are shown in adults’ responses.

The CAEPs measured from pre-lingually deafened CI users with background noise, i.e., speech-shaped noise at 10 dB SNR, are shown in Figure 13.

Figure 13 shows morphological changes of the P1-N1-P2 and the ACC with age. For onset responses, at 3 to 5 years, P1 is the dominant component with larger amplitude. At 6 to 8 years, P1 is still the only present peak, but with smaller amplitude. At 9 to 11 years, the N1 peak begins to emerge. N1 and P2 peaks become dominant in the onset response at 12 to 14 years. The morphology of the onset response becomes adult-like at 15 to 17 years. For ACC responses, P1 is the only component and is large in amplitude from 3 to 11 years. The N1-P2 peaks are evident at 12 to 14 years, and the amplitude grows at 15 to 17 years. However, the N1-P2 peak-to-peak amplitude appears to be small in the 18-year-olds’ and pre-lingually deafened adults’ grand average waveforms.

Summary statistics of peak latencies and N1–P2 amplitudes for CAEPs recorded from early-implanted, pre-lingually deafened CI users in noise are provided in Supplementary Table S7.

## 4 Discussion

This study aimed to explore the developmental trajectories of two types of cortical auditory evoked potentials—onset responses and the acoustic change complex—in normal hearing listeners and cochlear implant users across a wide age range, from young children to adults. Using identical procedures, the study successfully recorded these CAEPs under both quiet and noisy listening conditions. The findings provide valuable insights into the maturation of the central auditory system, highlighting that cochlear implants can effectively support its development in children who were born deaf and implanted early.

The developmental patterns of CAEPs in CI children are similar to those of NH listeners in quiet conditions, with both onset and ACC responses reaching comparable maturation levels to NH adults in quiet environments. However, ACC responses are more affected by noise than onset responses, particularly in CI users.

The results of this study have several clinical implications. First, the ability of cochlear implants to support the typical development of cortical auditory responses, as evidenced by similar developmental patterns in CI users and NH listeners, underscores the importance of early implantation. Early intervention maximizes the benefits of neuroplasticity, leading to better auditory and speech outcomes in children with congenital deafness.

Second, the significant impact of noise on auditory responses, particularly ACC responses, highlights the need for improved CI technology and auditory training programs that enhance noise resilience. Utilizing hearing assistive technologies, such as remote microphones, and developing strategies to improve sound discrimination in noisy environments could greatly benefit CI users, enhancing their daily communication and quality of life.

While this study suggests that cochlear implants support the development of the central auditory system in children, it does not directly compare auditory responses in children implanted before versus after the critical age of 3.5 years. Previous research indicates that early implantation maximizes neuroplasticity and supports typical auditory development. However, future studies should explore this further by comparing responses in children implanted before and after 3.5 years, matched for years of device usage. Such research could provide deeper insights into the specific benefits of early implantation and the influence of timing on central auditory system maturation.

### 4.1 Developmental patterns of P1-N1-P2 and ACC responses

In this study, using long-duration speech-like sounds, onset and ACC responses were successfully obtained from NH listeners and early-implanted (implanted before 3.5 years), pre-lingually deafened CI users, from 3-year-olds to adults, in both quiet and noisy listening conditions. The morphology, latency, and amplitude of the onset and ACC responses varied with age. The ACC followed similar developmental patterns to those of the onset response but matured later. P1 latency decreased significantly with age for both onset and ACC responses. These developmental patterns were consistent between NH listeners and CI users in both quiet and noisy conditions.

Previous research has primarily focused on the onset P1-N1-P2 response to investigate age-related changes. Findings indicate that P1 latency decreases with age over the first two decades of life in both NH listeners and CI users, particularly in those implanted before 3.5 years of age (Ponton et al., 1996a, 1996b; Sharma et al., 2002; Dorman et al., 2007). This suggests similar developmental patterns in both groups. Our results of the onset P1-N1-P2 complex in quiet conditions in both NH and CI groups agree with those of previous studies.

Previous CAEP development studies have used brief stimuli to elicit the onset P1-N1-P2 response alone. In this study, an 800-ms-long stimulus using two vowels was constructed to elicit both the onset and the ACC. The primary change appeared in F2 of the two vowels from about 1,200 Hz to 2,300 Hz, or vice versa. This stimulus was presented at a slow rate, about one stimulus every 3 s. Both the P1-N1-P2 complex and the ACC were successfully recorded from both NH and CI children. Results show that the onset P1 latency data from the current study fits well with the regression reported in previous studies (Sharma et al., 2002).

The highlight of the findings is the developmental patterns of ACCs. The ACC P1 latency decreases with age in both NH and CI listeners at the same rate. The ACC P1 latency is significantly longer than the onset P1 latency in both NH and CI groups. The ACC experiences the same morphologic changes but with later development than those of the onset response. In NH listeners, the ACC matures later than the onset response in quiet. Similar developmental patterns are seen in CI users, but with a slight delay compared to NH listeners. The onset response correlation data in NH listeners and CI users suggest a late maturation of the onset response in CI users by about 3 years when compared to NH listeners (Ponton et al., 1996a, 1996b).

While the current studies do not include performance measures, recent studies have explored the relationship between the P1 latency of cortical auditory evoked potentials in children with cochlear implants and their behavioral measures. For example, Frånlund et al. (2023) investigated this relationship and found that P1 latency is negatively correlated with language development among their youngest patients fitted with CIs. Specifically, shorter P1 latencies were associated with better language outcomes, suggesting that P1 latency might be a useful clinical tool to assess the maturation of central auditory pathways. Wang et al. (2023) studied P1 latency and the latency of mismatch negativity (MMN), which is related to discrimination ability, in CAEPs. They found that these latencies were negatively correlated with the duration of CI usage, meaning shorter latencies were associated with longer CI use. Additionally, these latencies were negatively correlated with speech perception, indicating that shorter latencies were linked to better speech perception outcomes, as measured by the LittlEARs^®^ Auditory Questionnaire, Categories of Auditory Performance, Speech Intelligibility Rating Scale, Infant-Toddler Meaningful Auditory Integration Scale, and Meaningful Use of Speech Scale scores. Studies with the ACC have also shown relationships between the ACC and performance measures. For instance, van Heteren et al. (2022) demonstrated that in both unilaterally and bilaterally deaf subjects, the N1 latency and N1-P2 amplitude of the CI ears correlated with speech perception and frequency discrimination, with shorter latencies and larger amplitudes indicative of better speech perception and frequency discrimination. Similarly, McGuire et al. (2022) examined the relationship between ACC metrics and cochlear implant speech outcomes, finding that larger N1′ amplitudes and shorter latencies were associated with better speech perception scores. Their findings further demonstrated that variability in cortical encoding of frequency changes, as reflected in ACC measures, explained 16–21% of the variability in CI users’ speech outcomes. These results emphasize the potential of using ACC metrics alongside CAEP measures like P1 and MMN latencies to predict auditory and language performance in CI users.

### 4.2 Impact of noise on auditory responses

Previous studies have successfully measured the ACC in CI users, demonstrating its sensitivity to various changes in auditory stimuli. For example, ACCs have been elicited by switching from one electrode to another (Brown et al., 2008; He et al., 2014; Kim et al., 2009; Mathew et al., 2017; Scheperle and Abbas, 2015), by spectral ripple noise (Won et al., 2011; Scheperle and Abbas, 2015; Brown et al., 2017), and by amplitude modulation changes (Jeon et al., 2025). ACCs have also been elicited by speech sounds (Martin et al., 2008; Brown et al., 2015; Friesen and Tremblay, 2006) and musical notes (Brown et al., 2017; Jeon et al., 2023, 2025). These findings show that the ACC can be used as a tool for assessing auditory discrimination in CI users under various stimuli and conditions.

Our study demonstrated that both the P1-N1-P2 and ACC responses were significantly degraded in the presence of background noise, characterized by smaller amplitudes and longer latencies. These effects delayed the development of the onset and ACC responses in noise for both NH and CI groups and were observed across all age groups. The finding that ACC responses were more affected by noise than the onset P1-N1-P2 responses supports our hypothesis and highlights the vulnerability of complex auditory processing to environmental interference.

The study documents the developmental patterns of CAEPs in background noise conditions. P1 latency decreases with age for the onset and the ACC in both NH and CI listeners. Both responses are affected by background noise, leading to delayed development compared to quiet conditions. N1-P2 amplitudes are significantly smaller in noise for both onset and ACC responses. The delayed development in noise is more pronounced for the ACC, especially in CI users. The ACC in CI listeners remain moderately correlated with NH adults after 14 years of age in noise conditions. Both pre-lingually and post-lingually deafened CI adults have significantly smaller ACC N1-P2 amplitudes than NH adults in noise (Sharma et al., 2002).

The pronounced impact of noise on ACC responses, particularly in CI users, underscores the additional challenges they face in noisy environments. Despite technological advancements in cochlear implants, CI users continue to experience difficulties with sound discrimination in the presence of background noise, affecting their communication abilities and overall auditory experience. These data outline developmental trends in NH listeners and CI users and provide an important basis for future studies of individual variations in development.

### 4.3 Neuroplasticity and changes in CAEPs in individual NH and CI users

This study, although not specifically designed to investigate individual changes over time, provides illustrative examples through the longitudinal data of two subjects. In NH listeners, neuroplasticity is suggested by the enhanced onset and ACC responses between the ages of 14 and 18, with a trend toward larger N1-P2 peak amplitudes observed at 18 years old compared to 14 years old, both in quiet and noisy conditions. While based on data from a single subject, these changes may reflect ongoing maturation and refinement of auditory cortical pathways, aligning with the natural developmental trajectory where repeated auditory experiences strengthen neural pathways, thus enhancing auditory processing abilities. Similarly, in CI users, neuroplasticity plays a critical roledue to the reliance on electrical stimulation to develop auditory pathways. The observed changes in ACC responses in one CI subject between the ages of 12 and 14, with a more adult-like morphology and the emergence of the N1-P2 response in noise at 14 years old, which was absent at 12 years old, illustrate the potential plasticity of the auditory system in CI users. These findings underscore the importance of consistent auditory input from the CI in promoting cortical maturation, consistent with prior research on neuroplasticity in auditory processing (Kral and Sharma, 2012; Gilley et al., 2008).

Further supporting the concept of neuroplasticity, Giedd et al. (1999) and Giedd (2004) provided a deeper understanding of anatomical changes in brain development. Their longitudinal pediatric neuroimaging studies identified significant developmental changes in brain anatomy from ages 4 to 20. They found linear increases in white matter and nonlinear changes in cortical gray matter, with a preadolescent increase followed by a postadolescent decrease. These changes were region-specific: frontal and parietal lobes peaked around age 12, the temporal lobe around age 16, and the occipital lobe continued increasing through age 20. MRI studies revealed dynamic anatomical changes throughout adolescence, with white matter increasing linearly across the major lobes and gray matter following an inverted U-shaped developmental course, peaking earlier in girls than boys. The dorsal lateral prefrontal cortex, crucial for impulse control, matures the latest, not reaching adult dimensions until the early 20s.

The rapid growth of a newborn brain further supports the concept of neuroplasticity. By 2–4 weeks, it reaches approximately 36% of the size of an adult brain (Knickmeyer et al., 2008). By 2 years, it achieves 80% of adult size, and by 5 years, it is about 90% of adult size (Dekaban, 1978; Knickmeyer et al., 2008). While brain size increases quickly in early life, neuronal structure and connections, influenced by both genetic and experiential factors, continue to mature into late adolescence and even into the twenties (Huttenlocher and Dabholkar, 1997; Giedd et al., 1999; Giedd, 2004; Lebel and Beaulieu, 2011; Khundrakpam et al., 2013). Brain development trajectories are nonlinear, with more significant changes occurring in young childhood compared to late adolescence and young adulthood (Lebel and Beaulieu, 2011). This period of young childhood is when the brain is most plastic and has the greatest capacity for reorganization (Zhang et al., 2002; Nakahara et al., 2004; Sale et al., 2009).

These combined findings underscore the dynamic nature of brain development and the significant role of neuroplasticity in both NH listeners and CI users. Understanding these developmental trajectories and the impact of auditory experiences on cortical maturation can inform clinical practices and interventions aimed at optimizing auditory outcomes in individuals with hearing impairments.

### 4.4 Limitations of the study

Limitations of this study include the small number of subjects, especially in the CI group, including young adult CI subjects. Additionally, children who were implanted young were defined as those implanted before 3.5 years of age. It was challenging to recruit early-implanted young adult CI users at the time of testing. However, as more children who were implanted at around 1 year old grow up, further studies should investigate if there are differences among early-implanted children across different implant ages.

The study assumed that post-lingually deafened adults have similar auditory development experiences to NH listeners, which may not be entirely accurate. Post-lingually deafened adults may experience auditory deprivation for varying durations before receiving cochlear implants, potentially leading to differences in cortical reorganization and auditory processing compared to NH listeners.

Additionally, only one type of background noise was used at a fixed level. Different types of noise or varying SNR levels may show different developmental patterns or significantly affect the CAEPs. Future studies with diverse stimuli and recording techniques could provide further insights.

This study focused on specific cortical responses, which do not fully characterize cortical sound processing. Moreover, the performance of the CI participants was not included. Future studies examining the developmental effects on CAEPs and their performance changes could provide deeper insights into the outcomes for CI users.

## 5 Conclusion

This study investigated the developmental trajectories of cortical auditory evoked potentials, specifically the P1-N1-P2 complex and the ACC, in both NH listeners and CI users. By examining these responses across various ages and listening conditions, the research underscores the significant role of neuroplasticity in auditory development. The findings indicate that early cochlear implantation facilitates the development of the central auditory system, allowing CI users to achieve maturation levels comparable to NH listeners in quiet conditions. However, the pronounced impact of background noise on ACC responses, especially in CI users, highlights the ongoing challenges in auditory discrimination in noisy environments. Additionally, the study suggests that the ACC can complement the onset response in documenting the development of the central auditory system, providing a measure for estimating perceptual performance in various age and listening groups. These results emphasize the need for continued advancements in CI technology and auditory training programs to enhance the listening experiences of CI users. Future research should further explore individual variations and the long-term developmental effects of early implantation and auditory experiences in diverse listening environments.

## Data Availability

The original contributions presented in the study are included in the article/Supplementary material, further inquiries can be directed to the corresponding author.
