# Supplementary material for: The effect of development on cortical auditory evoked potentials in normal hearing listeners and cochlear implant users
Source: Front Hum Neurosci. 2025 Oct 15;19:1473365. doi: 10.3389/fnhum.2025.1473365 (PMC12570083; doi:10.3389/fnhum.2025.1473365)

**SUPPLEMENTARY FIGURES AND TABLES**

**Supplementary Figure S1.**

**Grand Average Waveforms in Quiet and Noise Conditions for Normal-Hearing (NH) Listeners**


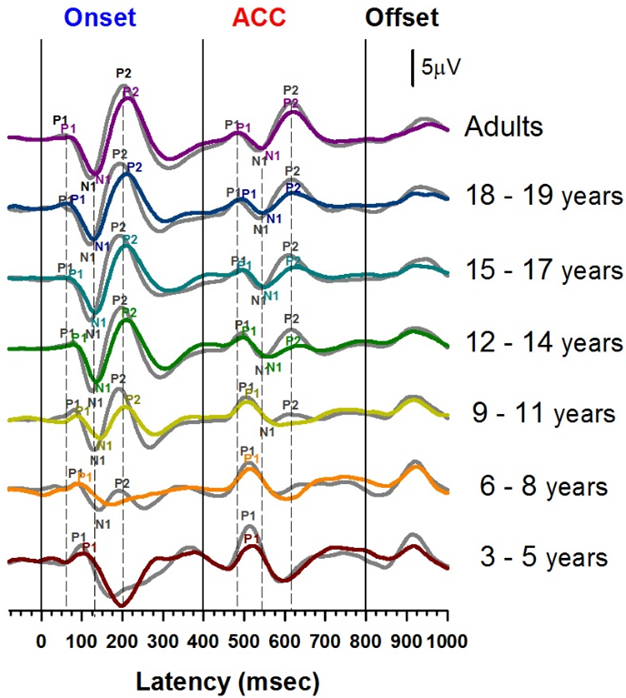


Grand-average cortical auditory evoked potentials (CAEPs) recorded in quiet and noise conditions for NH listeners. Gray lines represent responses in quiet; colored lines represent responses in background noise. Vertical lines at 0, 400, and 800 ms indicate stimulus onset, change, and offset, respectively. Dashed lines mark typical P1, N1, and P2 component latencies in adult responses under quiet conditions.

Adding background noise affected waveform morphology, particularly in younger children, reflecting delayed cortical maturation. At 6–8 years, N1 and P2 peaks are identifiable in the onset response in quiet, whereas P1 remains dominant in noise. From 9–14 years, N1 and P2 become more distinct with increasing auditory change complex (ACC) amplitude. In noise, N1–P2 peaks of the ACC are absent at 9–11 years and smaller than in quiet at 12–14 years.

**Supplementary Figure S2.**

**P1 Latency, N1 Detectability, and N1–P2 Amplitude in Quiet and Noise for NH Listeners**


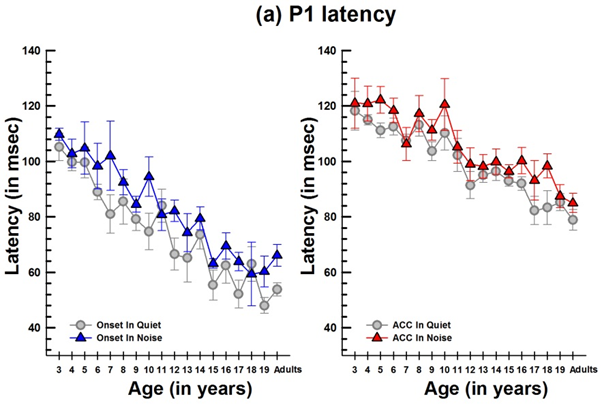


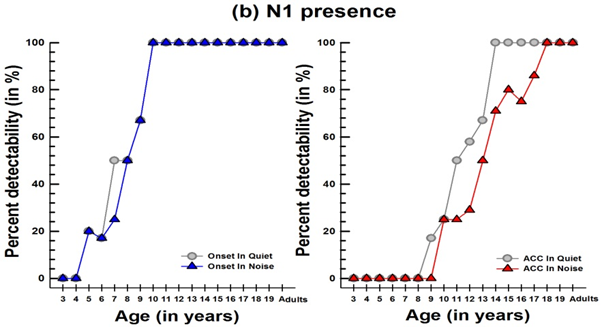


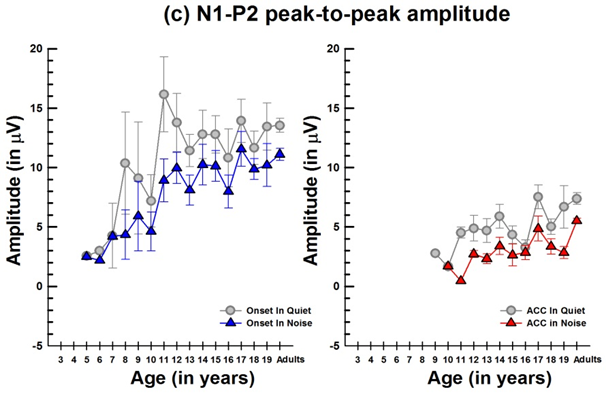


Figures show means ± SD for (a) P1 latency, (b) N1 detectability, and (c) N1–P2 peak-to-peak amplitude in NH listeners measured in quiet (gray) and noise (colored) conditions. The left panels show onset responses, and the right panels show auditory change complex (ACC) responses. Gray lines with dots represent data in quiet, and colored lines with dots represent data in noise.

In panel (a), P1 latency decreased significantly with age in noise for both the onset (p < 0.0001) and ACC (p < 0.0001) responses. The slope of P1 latency change was approximately 3 ms/year for the onset and 2 ms/year for the ACC. Age accounted for approximately 58% of the variance in onset P1 latency and 40% in ACC P1 latency (see Supplementary Table S2). The slopes differed significantly between onset and ACC latencies (t = 2.62, df = 168, p = 0.0095), suggesting distinct developmental patterns. ACC P1 latencies were significantly longer than onset P1 latencies in noise (mean difference = –12.83 ms, SD = 16.47, t = –12.83, df = 85, p < 0.0001). The slope of onset P1 latency with age did not differ between quiet and noise (α = 0.05), and no significant difference was found in the slope of ACC P1 latency between conditions, suggesting similar developmental patterns across listening environments. However, onset and ACC P1 latencies were both significantly shorter in quiet (onset: mean = –5.96 ms, SD = 12.75, t = –5.96, df = 85, p < 0.0001; ACC: mean = –5.82 ms, SD = 9.67, t = –5.82, df = 85, p < 0.0001).

In panel (b), N1 detectability increased systematically with age for both onset and ACC responses. The onset N1 was consistently identifiable in all participants by ~9 years of age, whereas the ACC N1 emerged later and reached full detectability by ~14 years. The ACC N1 component was not consistently present in all listeners until the late teenage years (approximately 18 years), indicating a delayed maturational pattern relative to the onset response.

In panel (c), the N1–P2 peak-to-peak amplitude measured in noise increased significantly with age only for the onset response (p < 0.0001), with a slope of approximately 0.6 µV/year. Age explained ~20% of the variance in the onset amplitude, while no significant linear relationship was observed for ACC amplitude. A paired t-test showed that the onset N1–P2 amplitude was significantly larger than the ACC amplitude (mean = 6.74 µV, SD = 2.75, t = 16.08, df = 42, p < 0.0001). The slope of onset amplitude change with age did not differ between quiet and noise (α = 0.05), suggesting comparable developmental patterns across conditions. When comparing listening conditions, amplitudes were significantly larger in quiet for both onset (mean = 3.06 µV, SD = 2.74, t = 9.42, df = 80, p < 0.0001) and ACC (mean = 2.10 µV, SD = 1.66, t = 8.29, df = 42, p < 0.0001) responses.

**Supplementary Figure S3.**

**Correlations Between Quiet and Noise Conditions in NH Listeners**


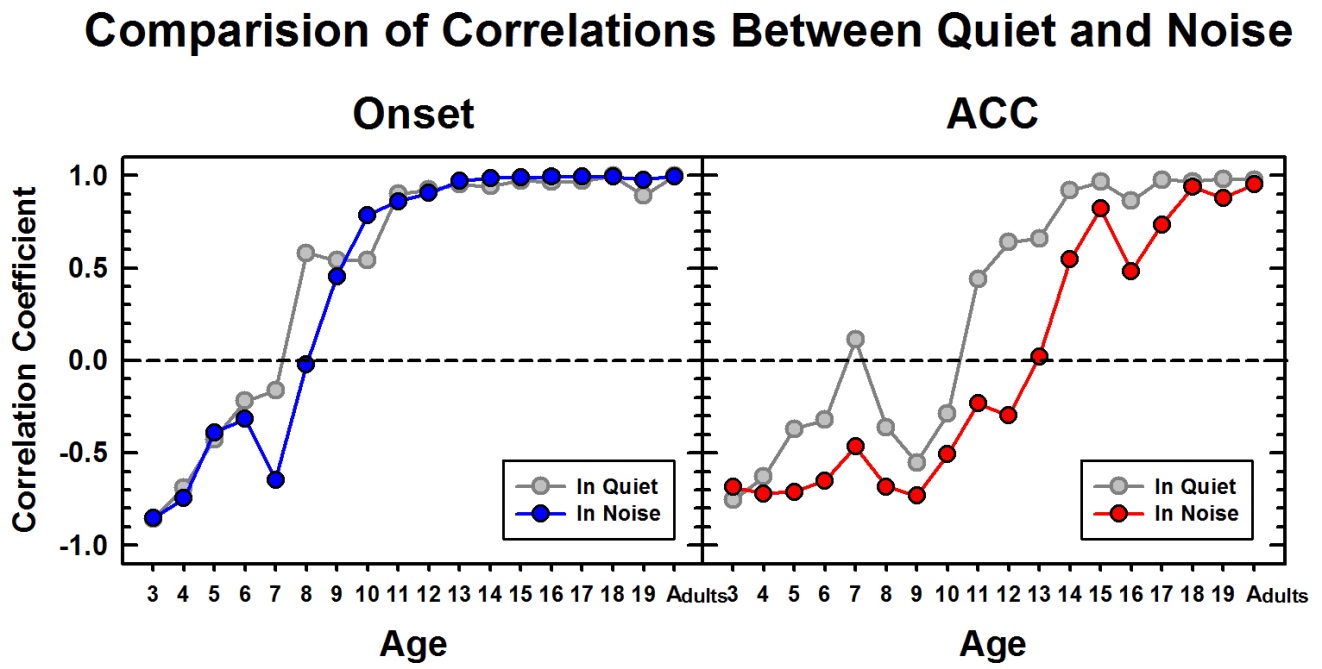


The left panel shows calculated correlation coefficients for the onset response in quiet and noise conditions. The right panel shows calculated correlation coefficients for the auditory change complex (ACC) in the same listening conditions. Gray lines with dots represent data obtained in quiet, and colored lines with dots represent data obtained in background noise.

Correlations with adult templates developed earlier in quiet than in noise, especially for the ACC. Noise produced poorer correlations at ages 7–9 for onset and extended developmental delays for the ACC, which reached adult-like correlation only in late adolescence.

**Supplementary Figure S4. Comparisons of CAEPs Among the Three Adult Listening Groups.**


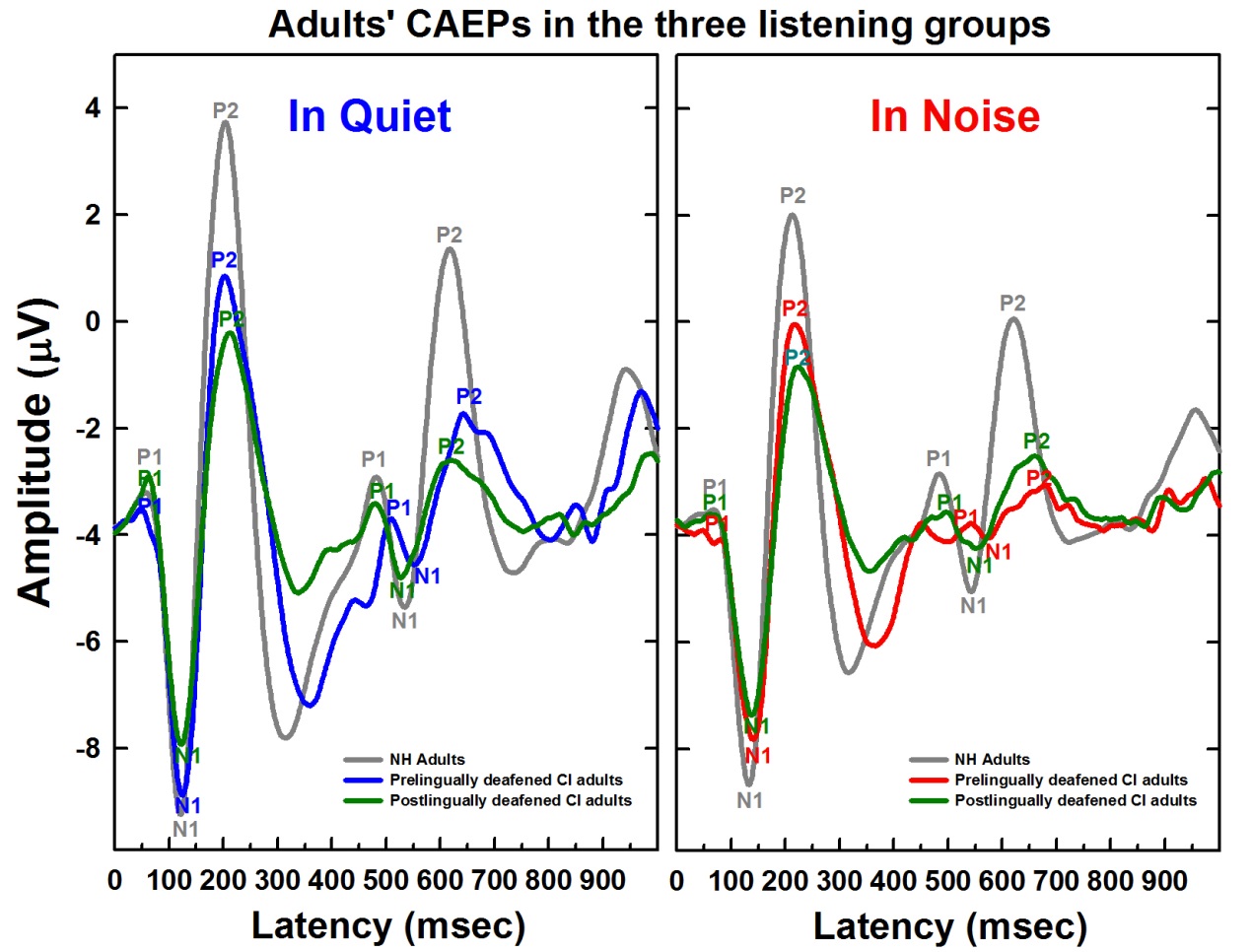


Grand average waveforms for NH adults (gray), post-lingually deafened CI adults (green), and pre-lingually deafened CI adults (blue = quiet; red = noise).

CAEP morphology was broadly similar across groups, though ACC amplitudes were smaller in both CI groups than in NH adults, particularly in noise. ANOVA results confirmed significant differences in ACC N1–P2 amplitudes between NH and both CI groups (*p* < 0.01), but not between the two CI groups. Detailed statistics are provided in Supplementary Table S5.

**Supplementary Figure S5. Development of N1 Component with Age in CI Users (Quiet Condition)**


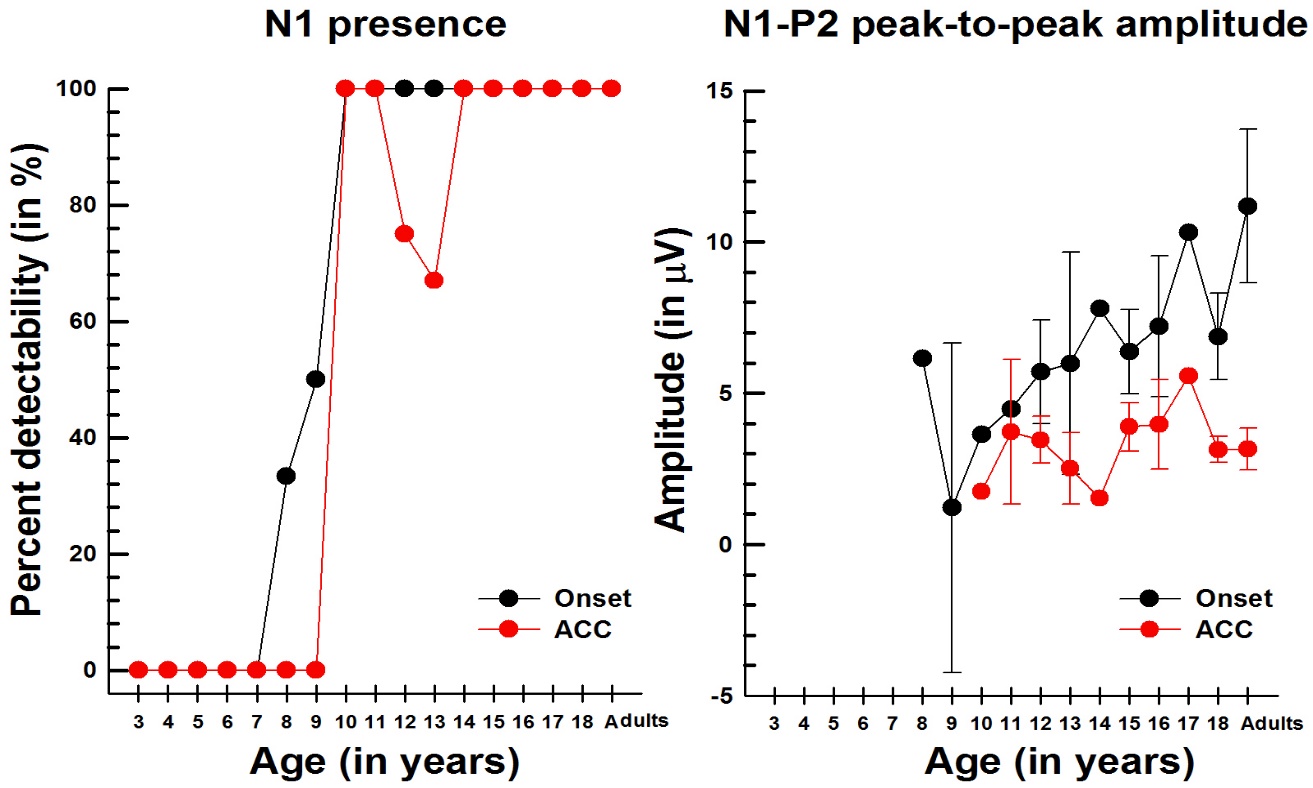


Left: N1 detectability; right: N1–P2 peak-to-peak amplitude versus age for onset (black) and ACC (red).

N1 emerged around 8 years for onset and was present in all CI users by 10 years; for the ACC, N1 remained variable until 13 years, reaching 100% presence by 14 years. The N1–P2 amplitude increased significantly with age for the onset response (p < 0.01; slope ≈ 0.6 µV/year). Onset amplitudes were significantly larger than ACC amplitudes (p < 0.0001).

**Supplementary Figure S6. Correlations Between CI Users’ and NH Adults’ CAEPs in Noise**


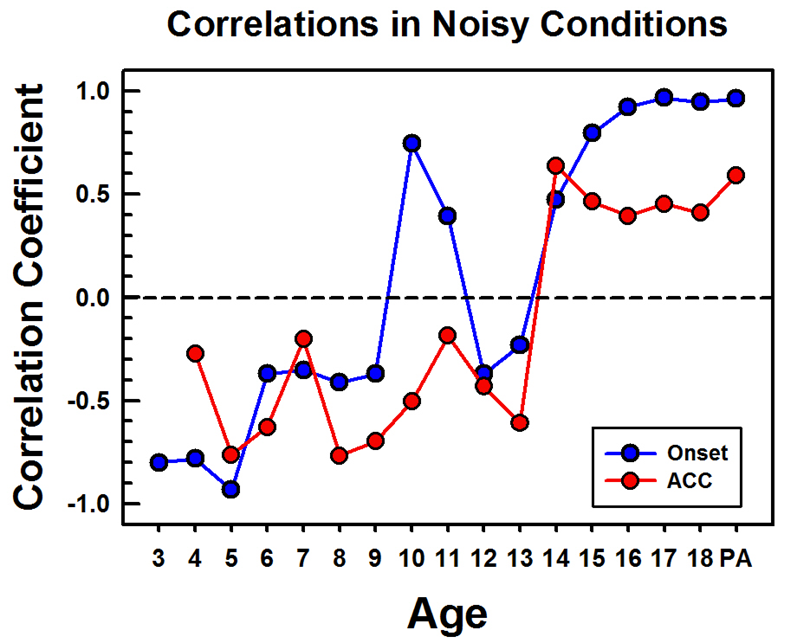


Correlation coefficients between CI users’ grand averages and NH adults’ waveforms (10 dB SNR). Blue = onset; red = ACC; PA = pre-lingually deafened adults.

Onset correlations plateaued around 16 years, while ACC correlations improved after 14 years but remained moderate in late teens and pre-lingually deafened adults.

**Supplementary Figure S7. Grand Average Waveforms in Quiet and Noise Conditions for CI Users**


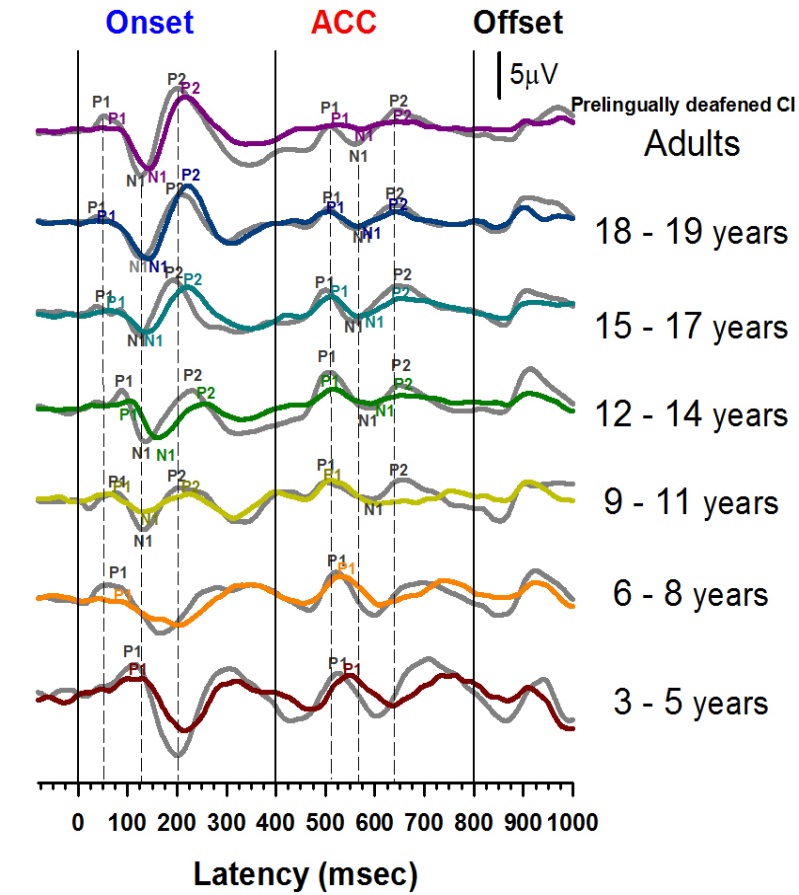


CAEPs recorded in quiet (gray) and noise (colored) conditions. Vertical lines mark onset, change, and offset; dashed lines indicate typical adult P1, N1, and P2 peaks.

Background noise reduced CAEP amplitudes and delayed components in CI users, similar to NH listeners. At 9–11 years, N1 and P2 peaks were visible in quiet but attenuated or absent in noise, particularly for the ACC. Pre-lingually deafened CI adults exhibited notably smaller ACC responses in noise.

**Supplementary Figure S8. P1 Latency, N1 Detectability, and N1–P2 Amplitude in Quiet and Noise for CI Users**

**
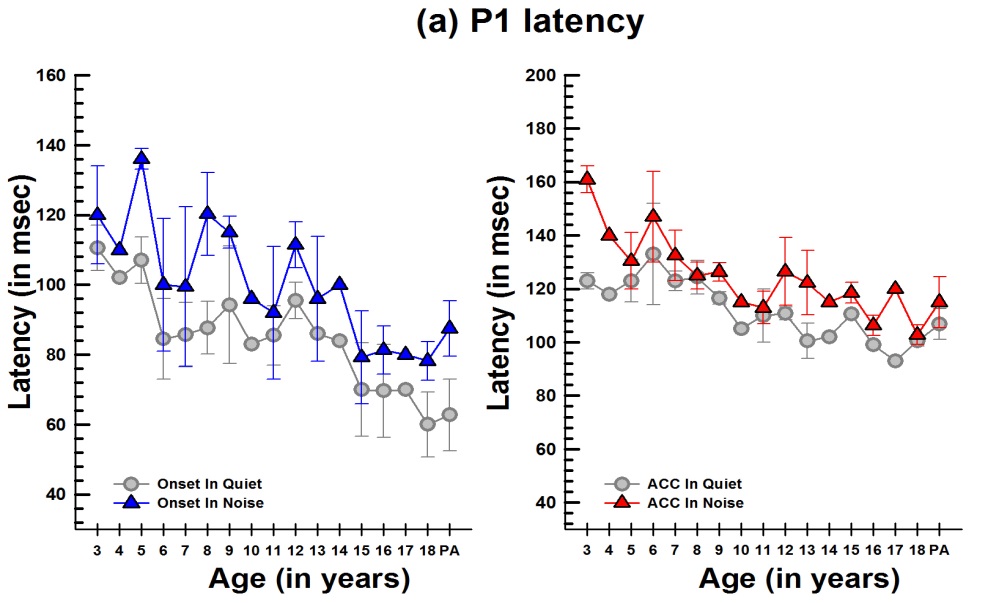
**

**
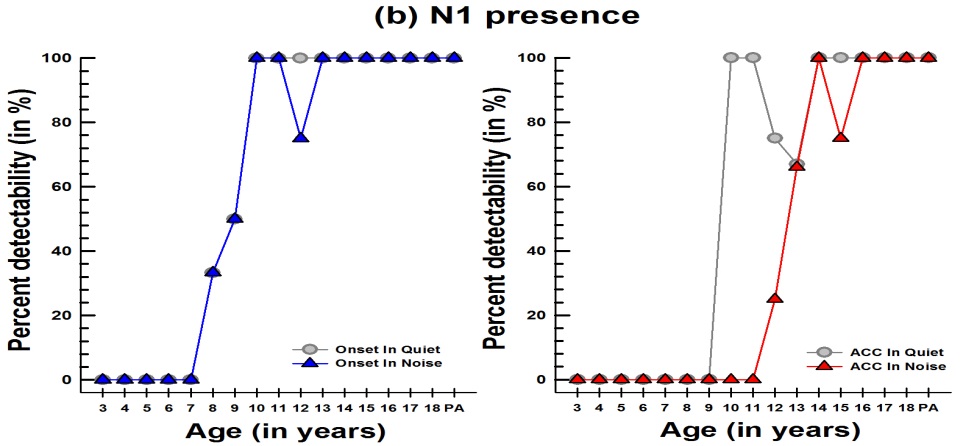
**

**
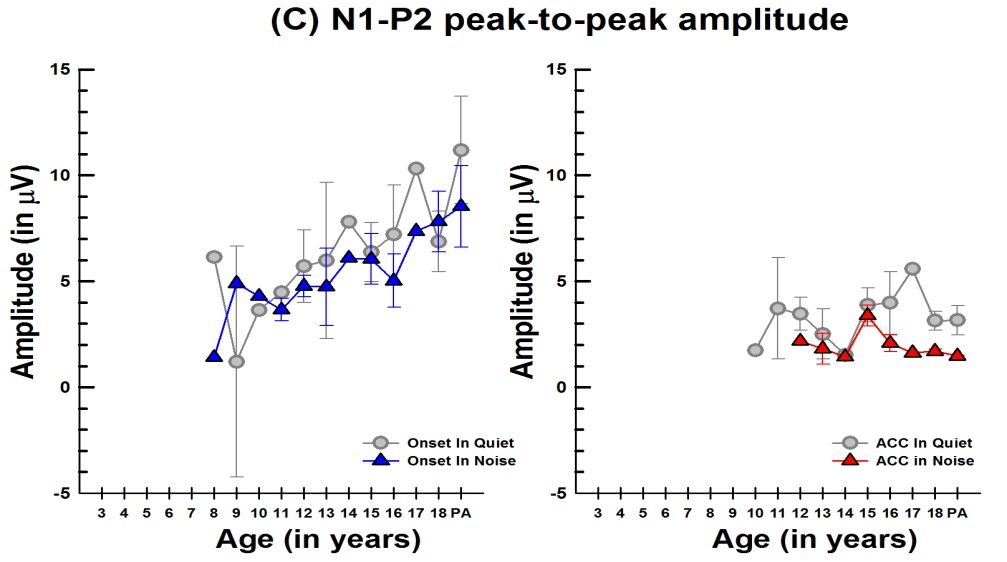
**

Figures show means ± SD for (a) P1 latency, (b) N1 detectability, and (c) N1–P2 peak-to-peak amplitude in early-implanted, pre-lingually deafened CI users measured in quiet (gray) and noise (colored) conditions. The left panels show onset responses, and the right panels show auditory change complex (ACC) responses.

In panel (a), P1 latency in noise decreased significantly with age for both onset (p = 0.0004) and ACC (p < 0.0001) responses. The slope of P1 latency change with age was approximately 3.0 ms/year for the onset and 2.7 ms/year for the ACC. Age explained about 28% of the variance in onset P1 latency and 44% in the ACC. The slopes of onset and ACC latency were not significantly different from each other at α = 0.05, suggesting similar developmental patterns. ACC P1 latencies were significantly longer than onset P1 latencies in noise (mean = –24 ms, SD = 24.26, t = –6.26, df = 39, p < 0.0001). The slope of onset P1 latency with age did not differ between quiet and noise (α = 0.05), and no significant difference was found for ACC latency slopes. This indicates that the developmental pattern of P1 latency was similar across listening conditions. However, both onset and ACC P1 latencies were significantly shorter in quiet than in noise (onset: mean = –15 ms, SD = 20.79, t = –4.93, df = 43, p < 0.0001; ACC: mean = –11 ms, SD = 12.89, t = –5.46, df = 43, p < 0.0001).

In panel (b), N1 detectability showed a clear developmental delay in noise for the ACC. With some variability, the onset N1 was present in all subjects by ~13 years, whereas the ACC N1 reached full detectability by ~16 years. This pattern suggests later cortical maturation for the ACC compared to the onset response, particularly under noisy listening conditions.

In panel (c), N1–P2 peak-to-peak amplitude measured in noise increased significantly with age only for the onset response (p = 0.0004). The N1–P2 amplitude increased by approximately 0.5 µV/year, with age explaining ~37% of the variance in onset amplitude. A paired t-test showed that onset N1–P2 amplitude was significantly larger than ACC amplitude (mean = 4.58 µV, SD = 3.03, t = 6.59, df = 18, p < 0.0001). The slope of onset N1–P2 amplitude with age did not differ significantly between quiet and noise at α = 0.05, indicating similar developmental trends across conditions. The onset amplitude did not differ significantly between quiet and noise (mean = 1.1 µV, SD = 2.91, t = 1.84, df = 23, p = 0.079). However, the ACC amplitude was significantly larger in quiet than in noise (mean = 1.5 µV, SD = 1.27, t = 5.12, df = 18, p < 0.0001).

**Supplementary Figure S9. Correlations Between Quiet and Noise Conditions in CI Users**

**
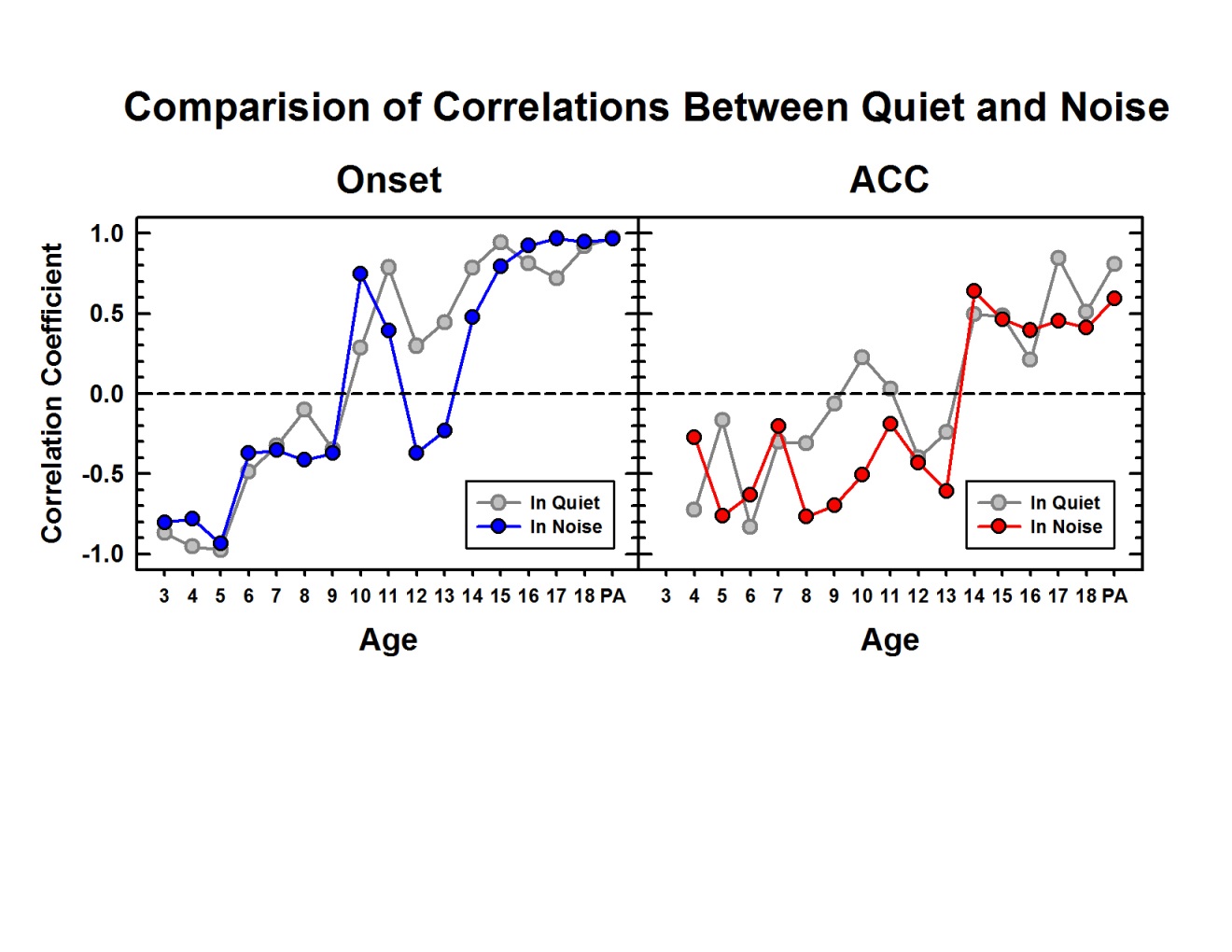
**

The left panel shows calculated correlation coefficients for the onset response in quiet and noise conditions, and the right panel shows those for the auditory change complex (ACC). Gray lines with dots represent data from quiet conditions, while colored lines represent data obtained with background noise. “PA” denotes pre-lingually deafened CI adults. All correlation coefficients were calculated between pre-lingually deafened CI children and normal-hearing (NH) adults’ grand average waveforms in both quiet and noise conditions.

Supplementary Figure S9 illustrates a delayed developmental pattern of correlation to adult waveforms in noise compared to quiet. This delay is more pronounced for the ACC. Adding background noise led to poorer correlations at ages 11–15 for the onset response, corresponding to ages when amplitude and latency of the ACC were most affected by noise. Correlation coefficients for onset responses approached 1.0 in both conditions, while ACC correlations in noise remained moderate even in adults.

**Supplementary Figure S10. Examples of Within-Subject Developmental Changes Over Time**


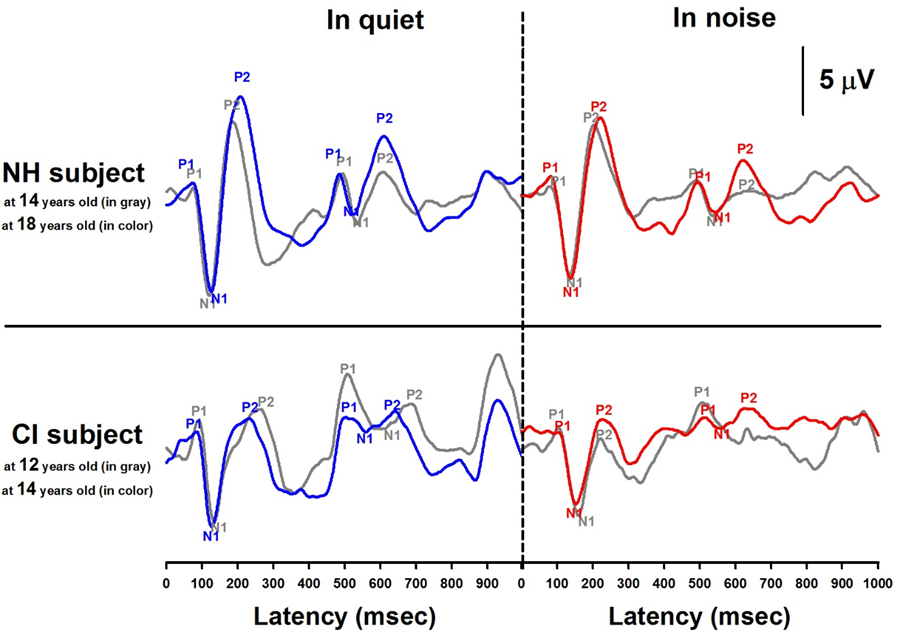


The top panel shows cortical auditory evoked potentials (CAEPs) from one normal-hearing (NH) listener recorded in quiet and noise conditions at ages 14 and 18 years. Gray solid lines indicate responses at 14 years, while blue and red lines indicate responses at 18 years in quiet and noise, respectively. The bottom panel shows CAEPs from one pre-lingually deafened cochlear implant (CI) user tested at ages 12 and 14 years, with the same color coding.

Although this study was not designed for longitudinal analysis, these two cases illustrate within-subject developmental changes. The NH listener demonstrated enhanced onset and auditory change complex (ACC) amplitudes with age, particularly in noise. At 18 years, N1–P2 peaks were more clearly defined compared to 14 years. The CI user exhibited progressive ACC maturation between 12 and 14 years: the P1-dominant morphology observed at 12 years evolved into an adult-like N1–P2 complex at 14 years, especially in noise conditions. These examples highlight ongoing cortical maturation in both NH and CI listeners during adolescence.

**SUPPLEMENTARY TABLES**

**Supplementary Table S1. NH Adults’ CAEPs: Quiet vs. Noise**


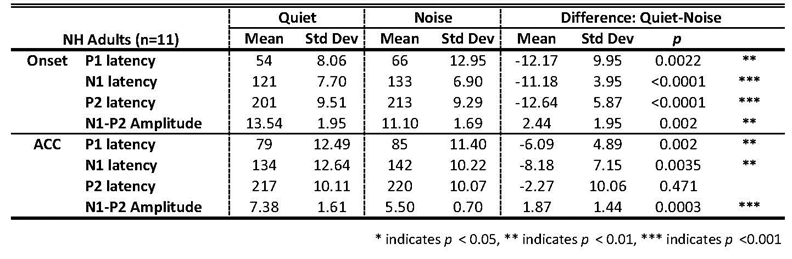


**Supplementary Table S2. Peak Latencies and N1-P2 Amplitudes for NH Listeners in Quiet**


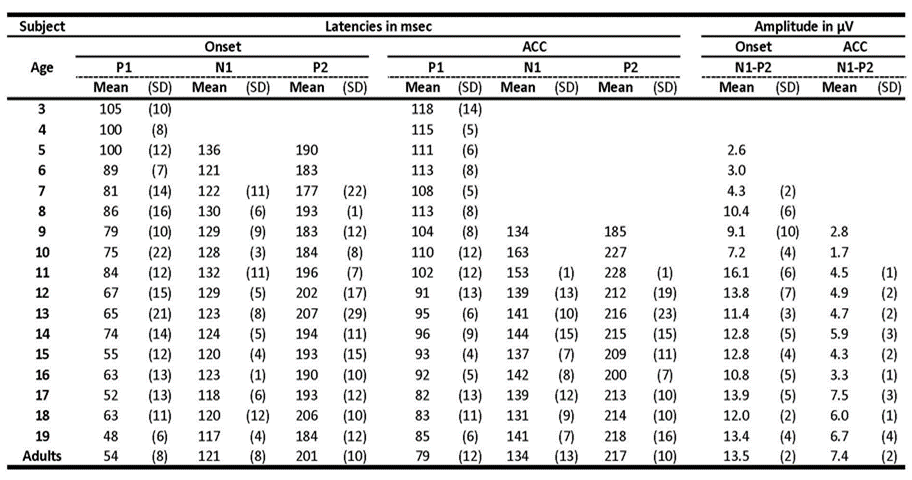


**Supplementary Table S3. Linear Regression of P1 Latency and N1-P2 Amplitude with Age in NH Listeners (Quiet Condition)**
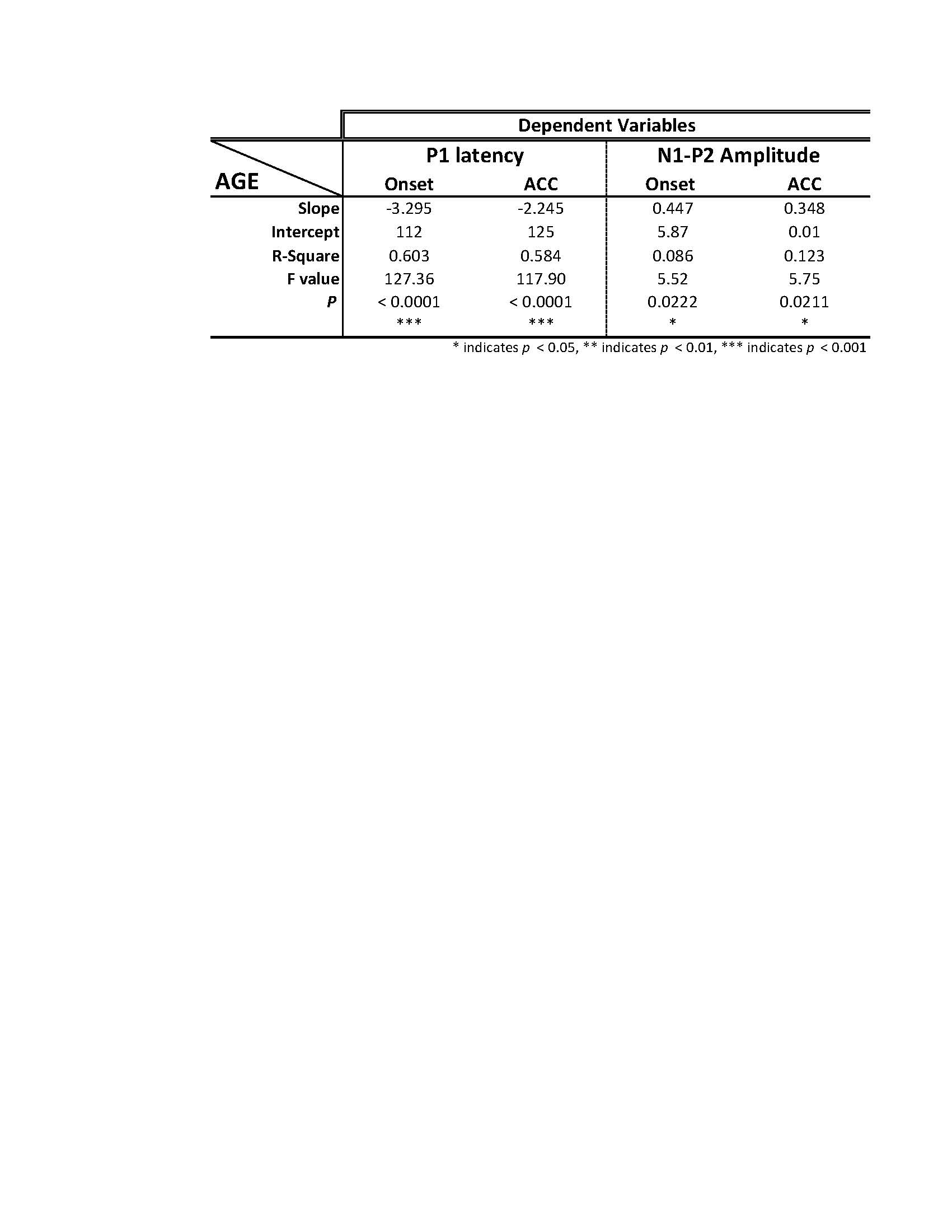


**Supplementary Table S4. Peak Latencies and N1-P2 Amplitudes for NH Listeners in Noise**


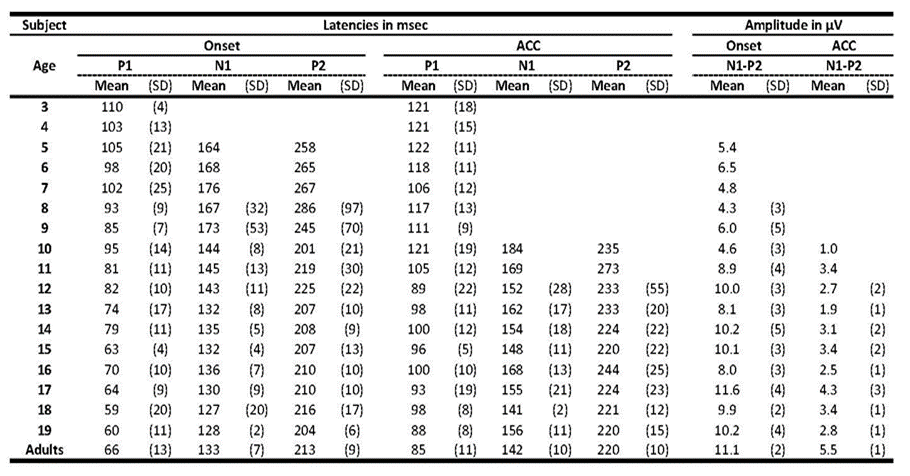


**Supplementary Table S5. Differences in Latencies and Amplitudes Among Adult Groups in Quiet and Noise**


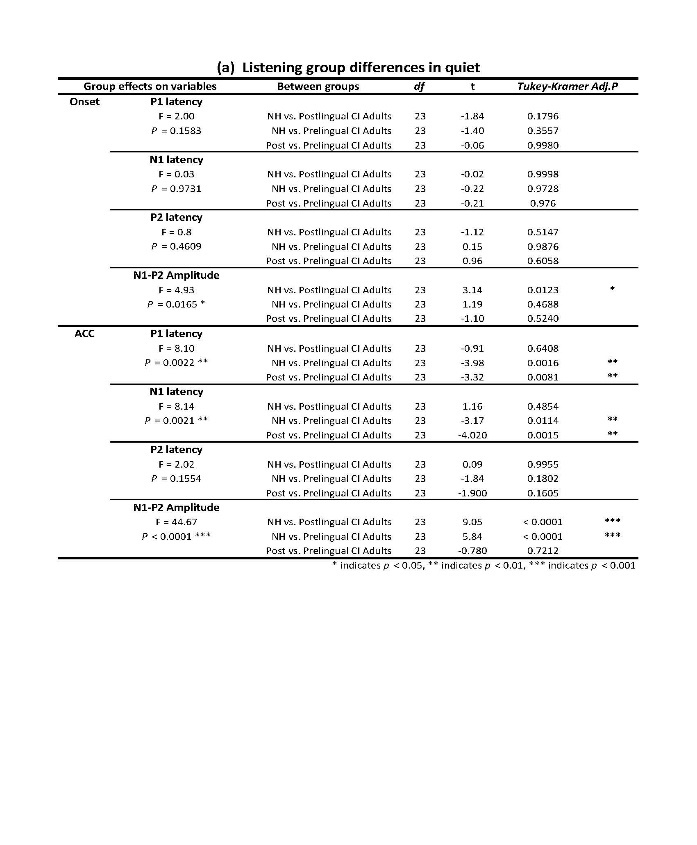

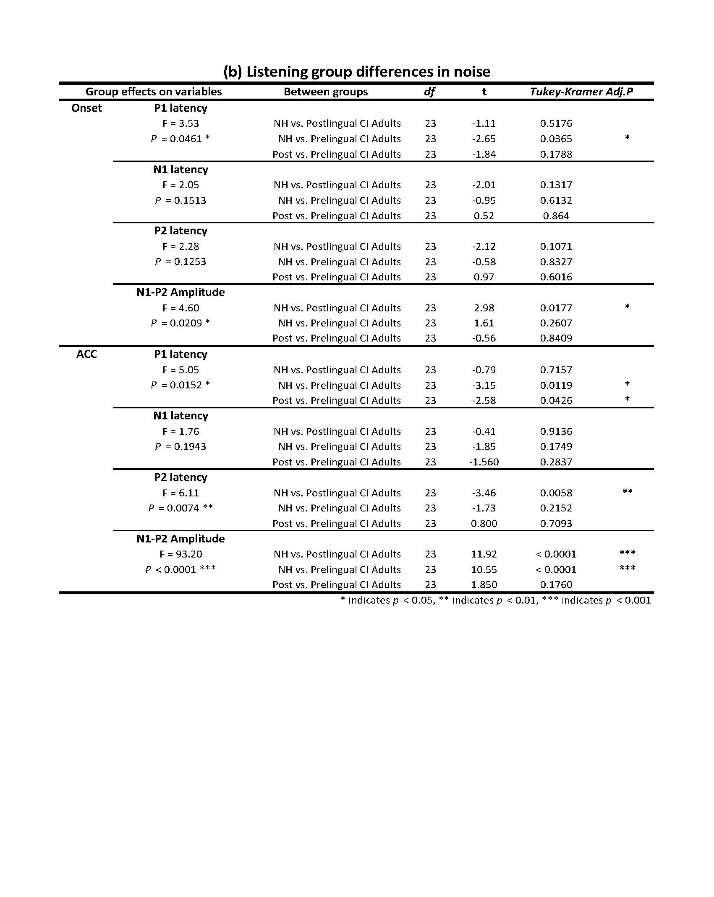


**Supplementary Table S6. Peak Latencies and N1-P2 Amplitudes for CI Users in Quiet**


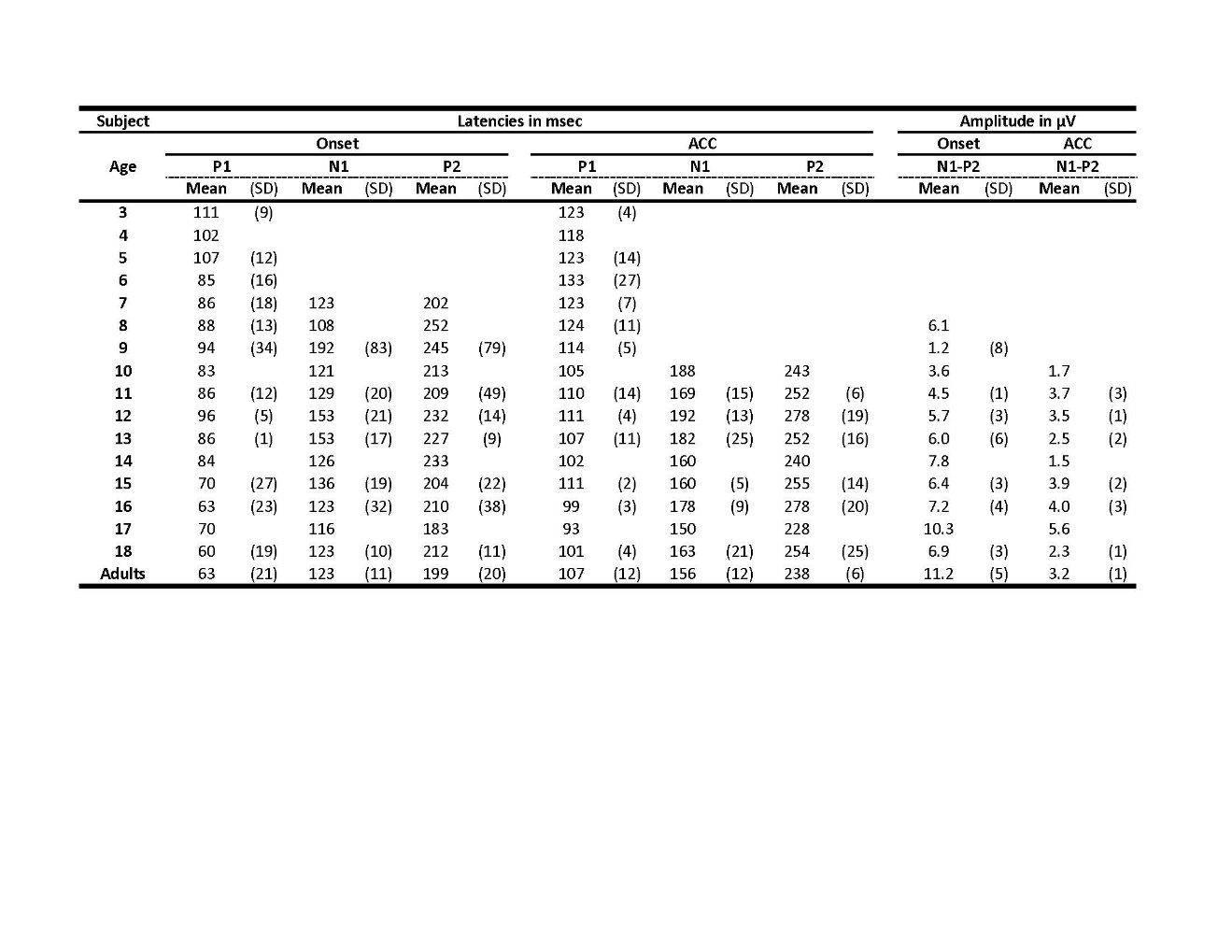


**Supplementary Table S7. Peak Latencies and N1-P2 Amplitudes for CI Users in Noise**


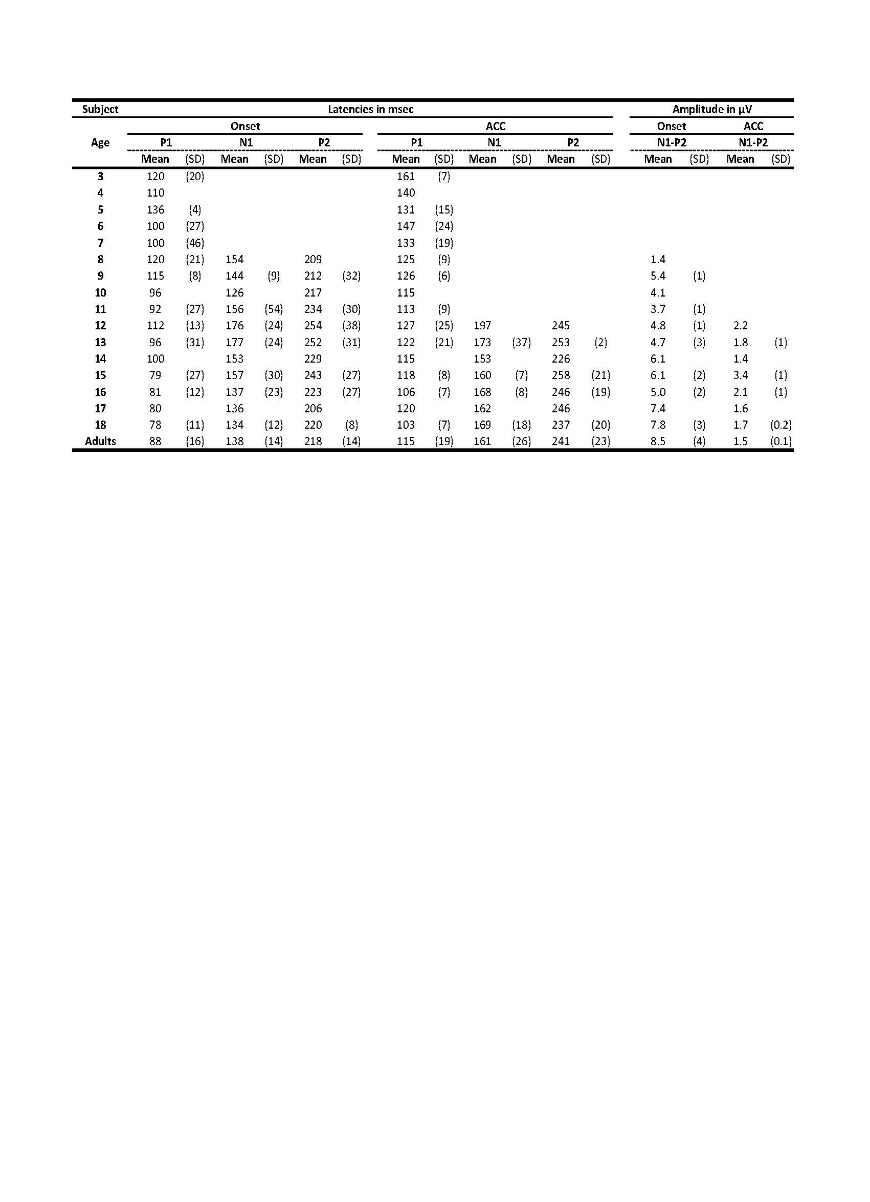

Supplement: Supplementary file 1 [file Supplementary_file_1.docx]
